# Engineering protected cavity-QED interactions through pulsed dynamical decoupling

**DOI:** 10.1038/s41534-025-01143-5

**Published:** 2025-11-21

**Authors:** I. Arrazola, P. Bertet, Y. Chu, P. Rabl

**Affiliations:** 1 EHU Quantum Center and Department of Physics, University of the Basque Country UPV/EHU, Bilbao, Spain; 2 Instituto de Física Teórica, UAM-CSIC, Universidad Autónoma de Madrid, Madrid, Spain; 3 Quantronics Group, SPEC, CEA Saclay, CNRS, Université Paris-Saclay, Gif-sur-Yvette, France; 4 Department of Physics, ETH Zürich, Zurich, Switzerland; 5 Walther-Meißner-Institut, Bayerische Akademie der Wissenschaften, Garching, Germany; 6 Technische Universität München, TUM School of Natural Sciences, Physics Department, Garching, Germany; 7 Munich Center for Quantum Science and Technology (MCQST), Munich, Germany

**Keywords:** Quantum information, Quantum optics

## Abstract

We study a generic cavity QED setup under conditions where the coupling between the two-level systems and a single bosonic mode is significantly degraded by low-frequency noise. To overcome this problem, we identify pulsed dynamical decoupling strategies that suppress the effects of noise while still allowing for a coherent exchange of excitations between the individual subsystems. The corresponding pulse sequences can be further designed to realize either Jaynes-Cummings, anti-Jaynes-Cummings, or Rabi couplings, as well as different types of cavity-mediated interactions between the two-level systems. A detailed analysis of the residual imperfections demonstrates that this decoupling strategy can boost the effective cooperativity of the cavity QED system by several orders of magnitude and improve the fidelity of quantum-technologically relevant operations accordingly.

## Introduction

The Jaynes-Cummings (JC) model describes the near-resonant coupling of a two-level system (TLS) to a single bosonic mode and for many decades it served as a prototypical toy model for studying light-matter interactions at the quantum level^1–3^. In recent years, the JC model has regained considerable interest in the context of quantum technologies, where it describes the basic processes relevant for generating non-classical photonic states^4^ or for realizing qubit-photon interfaces^5^. The cavity mode can also be used to implement long-range interactions between two or more TLSs, as it has already been demonstrated in a variety of systems ranging from optical cavity QED^6^ and trapped ions^7^ to superconducting circuits^8^ and solid-state spin qubits^9^. For all of these applications it is required that the coupling between the TLS and the bosonic mode is sufficiently strong in order to overcome the relevant decoherence processes in the system.

While in many of the originally considered cavity QED experiments with atoms and optical photons the decoherence rate of the TLS is mainly determined by the decay rate of the excited atomic state, this is not necessarily the case in other experimental platforms, where equivalent interactions are studied. Prominent examples include optical cavity QED systems with rare-earth dopants^10–13^ as well as spin ensembles^14–17^, individual impurity spins^18^ and gate-defined quantum dots^9,19–21^ coupled to microwave resonators. In those and many other systems of interest, spontaneous decay is often negligible compared to the dephasing rate ${\Gamma }_{\phi }=1/{T}_{2}^{* }$ associated with inhomogeneous broadening, low-frequency magnetic noise or other slow parameter drifts. For isolated TLSs, it is well-known that quasi-static shifts can be effectively suppressed using spin-echo techniques or more advanced pulsed dynamical decoupling (DD) schemes^22–26^. However, applying pulsed DD in cavity QED systems presents unique challenges, as fast *π*-rotations also disrupt or cancel^27^ the coherent interaction between the TLS and the cavity mode. This hinders a straightforward adaption of DD techniques in such systems.

A possible way to overcome this problem is to continuously drive the TLS with a strong external field. The fast Rabi oscillations then average out any quasi-static energy shifts^28–33^, while still permitting a resonant interaction between the cavity mode and the resulting dressed qubit states^34^, or the realization of protected quantum gates^35–40^. However, this continuous DD technique comes with several practical limitations. In particular, it is often difficult to control the power of the driving field with sufficient accuracy^41,42^ and the application of a strong and continuous driving field can lead to undesired heating effects^43^. With few exceptions^37,44^ these complications have hindered a widespread use of continuous DD schemes so far, especially in cryogenic experimental setups.

In this paper, we present an alternative approach for protecting cavity QED systems against low-frequency noise, leveraging the well-established pulsed DD techniques for individual TLSs^45–47^. In particular, going beyond a specific sequence that has been previously proposed for this problem^48^, we present a general framework for identifying and characterizing sequences of fast *π*-rotations, which recover the JC model as an effective interaction, despite the fact that the system dynamics is repeatedly interrupted. Further, we find that different variants of these pulse sequences can be used to engineer effective anti-JC or Rabi-type interactions, which are not present in the original system. The same approach can also be applied to protect cavity-mediated interactions between two or more TLSs, with a similar flexibility in the design of the effective interactions through an appropriate choice of pulse parameters.

From a detailed analysis of these processes, we find that noise-induced errors can be systematically suppressed by increasing the number of applied *π*-pulses, *N*_*π*_. Specifically, for cavity-mediated quantum gates, the residual error scales as ${\mathcal{E}} \sim 1/{({\mathcal{C}}{N}_{{\rm{\pi }}})}^{4/5}$, where ${\mathcal{C}}$ is the cooperativity of the bare cavity QED system. This almost linear improvement can be used to substantially boost the fidelity of cavity-mediated gate operations in existing setups, but also to enable the experimental realization of new cavity QED platforms that have so far been hindered by the presence of excessive noise. More generally, the techniques described in this paper extend previous schemes for pulsed Hamiltonian engineering for interacting spin systems^49–53^ to a more general set of spin-boson-type models, as relevant in quantum optics and various areas of solid-state physics.

## Results

### The noisy JC model

**Fig. 1:**
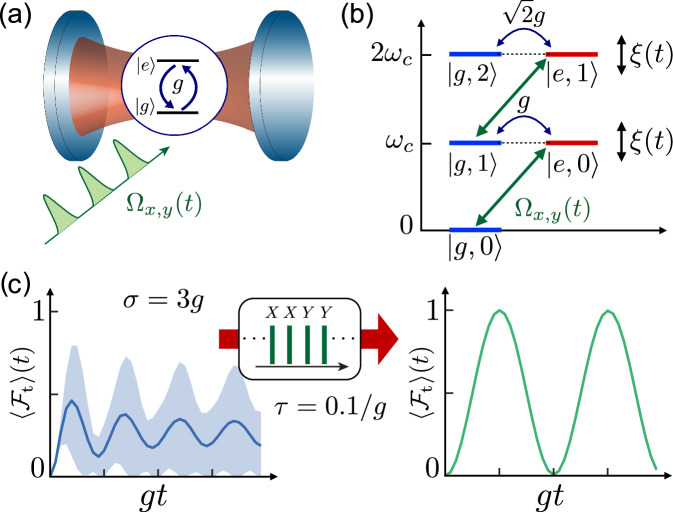
Protected vacuum Rabi oscillations. **a** Sketch of a cavity QED setup, where a TLS is coupled to a near-resonant bosonic mode. **b** Energy diagram of the relevant states of the JC model. In the presence of noise, the states $\left\vert e,n\right\rangle$ are shifted compared to the states $\left\vert g,n\right\rangle$ by a slowly fluctuating frequency *ξ*(*t*), which leads to dephasing of the bare TLS with a rate $1/{T}_{2}^{* }$. **c** Simulation of the vacuum Rabi oscillations for the case where the dephasing rate is comparable to the coupling strength (left panel). Under the same conditions, but interrupting this evolution by an appropriate sequence of *π*-pulses, the effect of the noise can be significantly suppressed while preserving the coherent oscillations between the TLS and the cavity mode (right panel). See text for more details.

We consider a generic cavity QED setup as shown in Fig. 1a, where a single TLS is coupled to a near resonant bosonic mode with frequency *ω*_*c*_ and annihilation and creation operators *a* and *a*^†^. The ground state $\left\vert g\right\rangle$ and the excited state $\left\vert e\right\rangle$ of the TLS are separated by a bare transition frequency *ω*_0_. In a rotating frame with respect to this frequency and under the validity of the rotating-wave approximation, the system is described by the JC Hamiltonian (*ℏ* = 1)

1 $${H}_{{\rm{JC}}}(t)=\Delta {a}^{\dagger }a+g({\sigma }_{+}a+{\sigma }_{-}{a}^{\dagger })+\frac{\xi (t)}{2}{\sigma }_{z},$$

where *g* is the coupling strength and *Δ* = *ω*_*c*_ − *ω*_0_ is the detuning. In Eq. (1), *ξ*(*t*) accounts for an additional unknown frequency shift of the TLS, which describes, for example, the effect of magnetic field fluctuations or other sources of low-frequency noise. For concreteness, we model *ξ*(*t*) in terms of an Ornstein-Uhlenbeck process^54^ with zero mean, 〈*ξ*(*t*)〉 = 0, and

2 $$\langle \xi (t)\xi (0)\rangle ={\sigma }^{2}{e}^{-t/{\tau }_{c}}.$$

Here *σ* and *τ*_*c*_ quantify the strength of the noise and its correlation time, respectively.

In this work we are primarily interested in the experimentally relevant regime of slow noise, *τ*_*c*_ ≫ *τ*_*π*_, in which case fast DD pulses with a duration *τ*_*π*_ can be applied to suppress the low-frequency components of the noise. For this purpose, we consider a total Hamiltonian of the form

3 $$H(t)={H}_{{\rm{JC}}}(t)+{H}_{{\rm{drive}}}(t),$$

where

4 $${H}_{{\rm{drive}}}(t)=\frac{{\Omega }_{x}(t)}{2}{\sigma }_{x}+\frac{{\Omega }_{y}(t)}{2}{\sigma }_{y}$$

accounts for external driving fields with switchable Rabi frequencies Ω_*x*_(*t*) and Ω_*y*_(*t*). These control fields are used to implement fast *π*-rotations of the TLS along the *x*-axis ("*X* pulse") or the *y*-axis ("*Y* pulse"), respectively.

For concreteness, in the following analysis we assume that the driving fields can be well-approximated by rectangular pulses, which are standard in experiments and provide the fastest *π*-pulses. These are described by

5 $${\Omega }_{\eta = x,y}(t)=\sum\limits_{i}\left(\pi +\delta {\theta }_{i}^{\eta }\right)\Theta (t-{t}_{i}^{\eta }).$$

Here the ${t}_{i}^{x}$ (${t}_{i}^{y}$) denote the times at which the individual *X* (*Y*) pulses are applied and the window function assumes a value of *Θ*(*t*) = 1/*τ*_*π*_ for *t* ∈ { − *τ*_*π*_/2, *τ*_*π*_/2} and *Θ*(*t*) = 0 otherwise. Therefore, for $\delta {\theta }_{i}^{x,y}=0$, the driving field implements a series of complete *π*-rotations of the form $X={e}^{-i{\sigma }_{x}\pi /2}$ and $Y={e}^{-i{\sigma }_{y}\pi /2}$. Finite values of $| \delta {\theta }_{i}^{x,y}| \ll \pi$ correspond to small under- or over-rotations that may arise from pulse imperfections. In addition, such pulse deviations can also be deliberately introduced in a deterministic manner to engineer specific effective interactions, as explained below.

In the absence of noise, the JC Hamiltonian *H*_JC_ preserves the total number of excitations and induces coherent oscillations between the states $\left\vert e,n-1\right\rangle$ and $\left\vert g,n\right\rangle$ with a photon-number dependent Rabi-frequency $2g\sqrt{n}$ on resonance [see Fig. 1b]. Specifically, by initializing the system in state $\left\vert \psi (0)\right\rangle =\left\vert e,0\right\rangle$, the state evolves as

6 $$\left\vert \psi (t)\right\rangle =C(t)\left\vert e,0\right\rangle -iS(t)\left\vert g,1\right\rangle ,$$

where $C(t)=\cos (\tilde{g}t)-i\sin (\tilde{g}t)\cos (\theta )$ and $S(t)=\sin (\tilde{g}t)\sin (\theta )$, with $\tilde{g}=\sqrt{{g}^{2}+{\Delta }^{2}/4}$ and a mixing angle given by $\tan (\theta )=2g/\Delta$. For *Δ* = 0 (*θ* = *π*/2) and a time *T*_t_ = *π*/(2*g*), the excitation is completely transferred from the TLS to the cavity mode, i.e., $\left\vert \psi ({T}_{{\rm{t}}})\right\rangle =\left\vert g,1\right\rangle$. Therefore, this process serves as a basic ingredient for preparing the cavity in a non-classical state or for realizing a qubit-photon interface.

This transfer quickly deteriorates, once random frequency fluctuations with *σ* ≳ *g* are introduced. For static noise, this effect can be understood from Eq. (6), by replacing the known detuning *Δ* by a random frequency offset *ξ*. Fig. 1c shows the resulting averaged state-transfer fidelity, $\langle {{\mathcal{F}}}_{{\rm{t}}}(t)\rangle$, along with its standard deviation (indicated by the shaded area). Here, ${{\mathcal{F}}}_{{\rm{t}}}(t)=| \langle g,1| \psi (t)\rangle {| }^{2}$ and 〈 ⋅ 〉 denotes the average over different noise realizations. For small noise strength, *σ*/*g* ≪ 1, the fidelity decays as (see Supplementary Note 1)

7 $$\langle {{\mathcal{F}}}_{{\rm{t}}}(t={T}_{{\rm{t}}})\rangle \approx 1-{\left(\frac{\sigma }{2g}\right)}^{2}.$$

This result confirms our basic intuition that the coupling strength *g* must considerably exceed the noise strength *σ*, in order to permit a coherent exchange of excitations. In many experimental settings, this regime cannot be reached^17^.

For the case of an uncoupled TLS, the effect of low-frequency noise can be efficiently suppressed by applying a fast *π*-rotation during half of the evolution. However, as can be seen from Eq. (6) and Fig. 1b, flipping the states $\left\vert g\right\rangle$ and $\left\vert e\right\rangle$ at a time *T*_t_/2 would populate the states $\left\vert g,0\right\rangle$ and $\left\vert e,1\right\rangle$, which live in different excitation-number subspaces and are decoupled from the target state $\left\vert g,1\right\rangle$ in the successive evolution. Therefore, while such a simple decoupling approach would reduce the impact of the noise, it would also completely spoil the state transfer.

### Dynamically protected vacuum Rabi oscillations

Let us now address how we can use DD pulses to suppress noise while preserving the coherent Rabi oscillations between the TLS and the cavity mode. To do so, we consider the dynamics of the noisy JC model, interrupted by a series of fast *π*-rotations. To identify an appropriate decoupling strategy, we change to a so-called toggling frame^55^ via the unitary transformation

8 $$\left\vert \psi (t)\right\rangle ={U}_{\pi }(t){e}^{-i\Delta t{a}^{\dagger }a}\left\vert \tilde{\psi (t)}\right\rangle .$$

Here, ${U}_{\pi }(t)={\mathcal{T}}{e}^{-i\mathop{\int}\nolimits_{0}^{t}ds{H}_{{\rm{drive}}}(s)}{| }_{\delta {\theta }_{i}^{x,y} = 0}$ describes the bare evolution of the TLS under the influence of the external driving field, assuming perfect *π*-pulses. In this new frame, the system evolves according to the Hamiltonian

9 $$\tilde{H(t)}={\tilde{H}}_{{\rm{int}}}(t)+\delta {\tilde{H}}_{{\rm{drive}}}(t)+\frac{\xi (t)}{2}{\tilde{\sigma }}_{z}(t),$$

where we have defined $\tilde{O(t)}={U}_{\pi }^{\dagger }(t)O{U}_{\pi }(t)$ and

10 $${\tilde{H}}_{{\rm{int}}}(t)=g\left[{\tilde{\sigma }}_{-}(t){a}^{\dagger }{e}^{i\Delta t}+{\rm{H.c.}}\right]$$

is the interaction Hamiltonian in the toggling frame. In Eq. (9), we have further included the term

11 $$\delta {\tilde{H}}_{{\rm{drive}}}(t)=\frac{\delta {\Omega }_{x}(t)}{2}{\tilde{\sigma }}_{x}(t)+\frac{\delta {\Omega }_{y}(t)}{2}{\tilde{\sigma }}_{y}(t)$$

to account for deviations from the complete *π*-rotations assumed in the definition of *U*_*π*_. Note that according to Eq. (5), $\delta {\Omega }_{x,y}(t)={\sum }_{i}\delta {\theta }_{i}^{x,y}\Theta (t-{t}_{i}^{x,y})$.

**Fig. 2:**
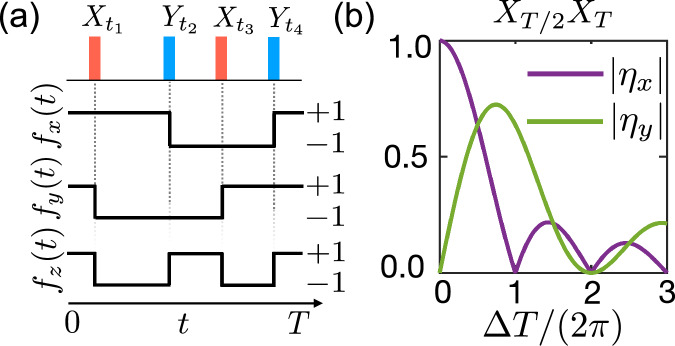
Modulation functions and effective parameters. **a** Plots of the modulation functions *f*_*x*,*y*,*z*_(*t*) for an example pulse sequence with four *π*-rotations (along both *x* and *y* axes) applied within a period *T.* **b** Dependence of the absolute values of *η*_*x*,*y*_ on the detuning *Δ* for the basic sequence *X*_*T*/2_*X*_*T*_.

While in all our numerical simulations a finite duration of the *π*-pulses is taken into account, for the following analytic considerations we restrict ourselves to instantaneous *π*-rotations and denote a specific pulse sequence by ${X}_{{t}_{1}^{x}}{Y}_{{t}_{1}^{y}}{Y}_{{t}_{2}^{y}}{X}_{{t}_{2}^{x}}\ldots \,$, etc. In this limit we find that

12 $${U}_{\pi }^{\dagger }(t){\sigma }_{k}{U}_{\pi }(t)={f}_{k}(t){\sigma }_{k},\qquad k=x,y,z,$$

where *f*_*k*_(*t*) = ±1. Specifically, as illustrated in Fig. 2a, the function *f*_*x*_(*t*) changes sign whenever a *Y* rotation is applied (i.e. at times ${t}_{i}^{y}$) and *f*_*y*_(*t*) changes sign whenever an *X* rotation is applied (i.e., at times ${t}_{i}^{x}$). For the *z* component we obtain *f*_*z*_(*t*) = *f*_*x*_(*t*)*f*_*y*_(*t*), which changes its sign after every pulse.

Given a pulse sequence with a total duration *T* that is short compared to *g*^−1^, we can use first-order perturbation theory to approximate the system evolution after this interval by a time-averaged effective Hamiltonian^55^

13 $${\tilde{H}}_{{\rm{eff}}}=\frac{1}{T}\mathop{\int}\nolimits_{0}^{T}ds\,\tilde{H(s)}={\tilde{H}}_{{\rm{int}}}^{{\rm{eff}}}+\delta {\tilde{H}}_{{\rm{drive}}}^{{\rm{eff}}}+{\tilde{H}}_{{\rm{noise}}}^{{\rm{eff}}}.$$

Here, the first term,

14 $${\tilde{H}}_{{\rm{int}}}^{{\rm{eff}}}=\frac{g}{2}\left[({\eta }_{x}{\sigma }_{x}-i{\eta }_{y}{\sigma }_{y}){a}^{\dagger }+{\rm{H.c.}}\right],$$

is the effective interaction Hamiltonian with complex parameters

15 $${\eta }_{x,y}=\frac{1}{T}\mathop{\int}\nolimits_{0}^{T}ds{f}_{x,y}(s){e}^{i\Delta s}.$$

This expression can be used to identify an appropriate set of decoupling pulses to obtain the desired effective light-matter coupling for this period. In Fig. 2b, the values of ∣*η*_*x*,*y*_∣ are plotted as a function of the detuning *Δ* for a simple DD sequence *X*_*T*/2_*X*_*T*_. As one can already see from this plot, the form of the effective interaction depends crucially on the value of *Δ*. For example, for *Δ* = 0, the interaction will be of the form ${\tilde{H}}_{{\rm{int}}}^{{\rm{eff}}} \sim {\sigma }_{x}(a+{a}^{\dagger })$, while for *Δ* = 4*π*/*T*, the interaction cancels out completely.

To extend this effective interaction to arbitrary times, we consider periodic pulse sequences with *f*_*k*_(*t* + *T*) = *f*_*k*_(*t*). Then, the effective parameters ${\eta }_{x,y}^{(n)}$ for the *n*-th time interval {*nT*, (*n* + 1)*T*}, fulfill

16 $${\eta }_{x,y}^{(n)}={e}^{in\Delta T}{\eta }_{x,y}.$$

By setting *ΔT* = 2*πm* + *Δ*_eff_*T*, where $m\in {\mathbb{Z}}$ and Δ_eff_ is a residual detuning, we can rewrite the effective interaction Hamiltonian for all time intervals as

17 $${\tilde{H}}_{{\rm{int}}}^{{\rm{eff}}}(nT)=\frac{{g}_{{\rm{eff}}}}{2}\left(\overrightarrow{n}\cdot \overrightarrow{\sigma }\,{a}^{\dagger }{e}^{i{\Delta }_{{\rm{eff}}}nT}+{\rm{H.c.}}\right).$$

Here *g*_eff_ = *ηg* is the effective coupling strength, $\overrightarrow{n}=({\eta }_{x},-i{\eta }_{y},0)/\eta$ and *η* = (∣*η*_*x*_∣ + ∣*η*_*y*_∣)/2.

In the limit *T* → 0 we can treat *t*_*n*_ = *nT* → *t* as a continuous time variable and ${\tilde{H}}_{{\rm{int}}}^{{\rm{eff}}}(t)$ then determines the exact dynamics of the cavity QED system. Even for finite *T*, Eq. (17) serves as a guideline to identify pulses that reproduce this dynamics to a very good approximation. When *η*_*x*_ = *η*_*y*_, ${\tilde{H}}_{{\rm{int}}}^{{\rm{eff}}}(t)$ corresponds to a JC model with detuning *Δ*_eff_ and coupling strength *g*_eff_. In contrast, when *η*_*x*_ = −*η*_*y*_, the effective Hamiltonian corresponds to a detuned anti-JC model, ${\tilde{H}}_{{\rm{int}}}^{{\rm{eff}}} \sim ({\sigma }_{+}{a}^{\dagger }+{\sigma }_{-}a)$, while the case *η*_*y*_ = 0, *η*_*x*_ ≠ 0 translates into a Rabi-coupling ${\tilde{H}}_{{\rm{int}}}^{{\rm{eff}}} \sim {\sigma }_{x}(a+{a}^{\dagger })$. Therefore, the identification of appropriate sequences of *X* and *Y* pulses not only allows us to recover the original JC interaction, but also to engineer effective interactions that were not present in the undriven system.

Apart from engineering the targeted effective coupling, a given pulse sequence must also fulfill its original purpose and protect the coupling against low-frequency noise and, ideally, also against pulse errors^56,57^. This requirement imposes the following additional conditions on the modulation functions *f*_*k*_(*t*). First of all, in order to cancel unwanted frequency shifts, we require

18 $${\tilde{H}}_{{\rm{noise}}}^{{\rm{eff}}}=\frac{1}{2T}\mathop{\int}\nolimits_{0}^{T}ds{f}_{z}(s)\xi (s){\sigma }_{z}=0,$$

which for quasi-static noise, *T* ≪ *τ*_*c*_, is achieved when

19 $${\gamma }_{z}=\frac{1}{T}\mathop{\int}\nolimits_{0}^{T}ds{f}_{z}(s)=0.$$

Second, small deviations from complete *π*-rotations will contribute with an effective Hamiltonian of the form

20 $$\delta {\tilde{H}}_{{\rm{drive}}}^{{\rm{eff}}}=\frac{{\upsilon }_{x}}{2}{\sigma }_{x}+\frac{{\upsilon }_{y}}{2}{\sigma }_{y},$$

where

21 $${\upsilon }_{\eta = x,y}=\frac{1}{T}\mathop{\int}\nolimits_{0}^{T}ds{f}_{\eta }(s)\delta {\Omega }_{\eta }(s)=\frac{1}{T}\sum\limits_{i}{f}_{\eta }({t}_{i}^{\eta })\delta {\theta }_{i}^{\eta }.$$

In the same way as quasi-static shifts are canceled out by sequences that fulfill *γ*_*z*_ = 0, sequences that fulfill

22 $${\gamma }_{x,y}=\frac{1}{T}\mathop{\int}\nolimits_{0}^{T}ds{f}_{x,y}(s)=0$$

will be robust against systematic rotation errors, $\delta {\theta }_{i}^{\eta }=\delta {\theta }^{\eta }\ne 0$. Note, however, that incomplete *π*-pulses can also be used on purpose to engineer deterministic Hamiltonian terms as in Eq. (20) with *υ*_*η*=*x*,*y*_ ≠ 0. This approach can be combined with the requirement *γ*_*x*,*y*_ = 0, for example, by applying alternating rotations with $\delta {\theta }_{i+1}^{\eta }=-\delta {\theta }_{i}^{\eta }$.

Let us now apply this general framework to identify specific pulse sequences that can be used to implement dynamically protected cavity QED Hamiltonians. We do so first for the case of the JC model and the anti-JC model, which only differ by a sign in the condition *η*_*x*_ = ± *η*_*y*_. Note that when, for example, only *X* pulses are applied we obtain *f*_*x*_(*t*) = 1 and either *η*_*y*_ = 0 for *m* = 0 or *η*_*x*_ = 0 for *m* > 0. Consequently, both *X* and *Y* pulses are required to engineer (anti) JC models with ∣*η*_*x*_∣ = ∣*η*_*y*_∣ ≠ 0. This explains the failure of the naive spin-echo pulse sequence discussed above. A second basic observation is that the functions *f*_*x*_(*t*) and *f*_*y*_(*t*) can only be periodic for an even number of *X* or *Y* pulses during the interval *T*. Therefore, the minimal sequence leading to an (anti) JC model must contain at least four (two *X* and two *Y*) pulses. When both *f*_*x*_(*t*) and *f*_*y*_(*t*) are periodic, *f*_*z*_(*t*) is periodic as well.

In the most general four-pulse sequence ${P}_{{t}_{1}}^{(1)}{P}_{{t}_{2}}^{(2)}{P}_{{t}_{3}}^{(3)}{P}_{{t}_{4}}^{(4)}$, we can still choose the times *t*_*i*_ and the order of the pulses *P* = *X*, *Y* to apply. For simplicity, here we focus on sequences of equally spaced pulses with a spacing *τ* = *T*/4. Since every pulse flips the sign of *f*_*z*_(*t*), this choice ensures that the condition *γ*_*z*_ = 0 is satisfied and the sequence protects against low-frequency noise. The condition *η*_*y*_ = ± *η*_*x*_, required for the (anti) JC model, then translates into the relation *f*_*y*_(*t*) = *f*_*x*_(*t* + *T*/2) for the toggling functions. From these assumptions it follows that *η*_*y*_ = (−1)^*m*^*η*_*x*_, where $m\in {\mathbb{Z}}$ is determined by the choice of the detuning *Δ* = 2*πm*/*T*.

The only four-pulse sequence that satisfies all those constraints is ${X}_{{t}_{1}}{X}_{{t}_{1}+\tau }{Y}_{{t}_{1}+2\tau }{Y}_{{t}_{1}+3\tau }$, where the time-offset *t*_1_ ∈ [ − *τ*, *τ*] is a free parameter. Note that here we allow for negative values of *t*_1_ to represent the sequence ${X}_{{t}_{1}+\tau }{Y}_{{t}_{1}+2\tau }{Y}_{{t}_{1}+3\tau }{X}_{{t}_{1}+4\tau }$. For this set of pulses we obtain the effective parameters ${\eta }_{y}=\eta {e}^{i{\phi }_{y}}$ and *η*_*x*_ = (−1)^*m*^*η*_*y*_, where

23 $$\eta =\frac{2}{m\pi }\sin \left(\frac{m\pi }{4}\right)$$

and *ϕ*_*y*_ = *πm*(8*t*_1_/*T* + 1)/4 − *π* for *m* ≠ 0, and *η* = 0.5 and *ϕ*_*y*_ = 0 for *m* = 0. The relative phase between *η*_*x*_ and *η*_*y*_ can be either 0 or *π*, such that the effective interaction has the form of a JC model,

24 $${\tilde{H}}_{{\rm{int}}}^{{\rm{eff}}}=\eta g\left({\sigma }_{-}{a}^{\dagger }{e}^{i\phi }+{\rm{H.c.}}\right),$$

with *ϕ* = *ϕ*_*y*_ when *m* is even and that of an anti-JC model,

25 $${\tilde{H}}_{{\rm{int}}}^{{\rm{eff}}}=\eta g\left({\sigma }_{+}{a}^{\dagger }{e}^{i\phi }+{\rm{H.c.}}\right),$$

with *ϕ* = *ϕ*_*y*_ + *π* when *m* is odd. Both models can be generalized to include a finite detuning *Δ*_eff_, as long as ∣*Δ*_eff_∣ ≪ 2*π*/*T*.

In Table 1 we summarize the values of the coupling coefficient *η* obtained for different $m\in {\mathbb{Z}}$, as well as the type of the resulting light-matter interaction. Interestingly, for values of *m* that are multiples of 4, the interaction cancels out completely. Such pulse sequences can be used, for example, to decouple the TLS from the cavity mode during idle times. In general, we find that larger values of ∣*m*∣ result in a smaller effective coupling strength. However, depending on the applied pulse sequence, a minimal value *m* ≠ 0 might be required to obtain a specific effective model. In this case, the initial detuning between the TLS and the cavity mode,

26 $$\Delta \simeq \frac{2\pi m}{T},$$

must match the pulse period. Importantly, this relation implies that by adjusting *Δ* accordingly, the pulse period *T* can be set to be arbitrarily short without reducing the coupling strength.

**Table 1:**
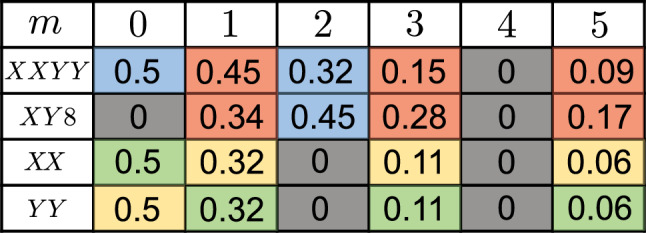
Summary of the numerical values of *η*, which determines the effective coupling strength, *g*_eff_ = *gη*, as obtained for different values of the detuning *Δ* = 2*πm*/*T* and different pulse sequences

The blue and red colors indicate that the corresponding pulse sequences realize JC and anti-JC interactions, respectively. The green and yellow colors refer to Rabi interactions of *σ*_*x*_-type and *σ*_*y*_-type instead.

Prior to this work, Groszkowski et al.^48^ suggested another four-pulse sequence to protect a resonant JC-coupling. In our notation, this sequence can be written as *X*_*T*/2_*X*_3*T*/4_*Y*_3*T*/4_*Y*_*T*_. It is not equally spaced and at time 3*T*/4 a combination of an *X* and a *Y* pulse is applied right after each other to implement an effective *Z*-rotation. This sequence satisfies all the conditions from above and realizes an effective JC-coupling with *g*_eff_ = *g*/2. For an ideal and noiseless systems, this sequence is very robust with respect to a finite spacing between the pulses, but typically it performs worse than the other sequences presented here under realistic conditions. This behavior can be understood from the error analysis presented in Supplementary Note 2.

A practical drawback of the four-pulse sequence from above is that it does not satisfy the conditions *γ*_*x*,*y*_ = 0, which are required to make it robust against pulse imperfections. Therefore, we now consider the XY8 sequence^56^${X}_{{t}_{1}}{Y}_{{t}_{1}+\tau }{X}_{{t}_{1}+2\tau }{Y}_{{t}_{1}+3\tau }{Y}_{{t}_{1}+4\tau }{X}_{{t}_{1}+5\tau }{Y}_{{t}_{1}+6\tau }{X}_{{t}_{1}+7\tau }$ with a pulse spacing *τ* = *T*/8 and − *τ* < *t*_1_ < *τ*. Again, we find that this sequence fulfills the symmetry condition *f*_*y*_(*t*) = *f*_*x*_(*t* + *T*/2), which results in an effective (anti) JC interaction. The coefficients in this case are ${\eta }_{y}=\eta {e}^{i{\phi }_{y}}$ and *η*_*x*_ = (−1)^*m*^*η*_*y*_ with

27 $$\eta =\frac{4}{\pi m}\sin \left(\frac{\pi m}{4}\right)\cos \left(\frac{5\pi m}{8}\right),$$

and *ϕ*_*y*_ = *πm*(16*t*_1_/*T* − 1)/8 + *π*(*m* + 1), while *η* = 0 for *m* = 0. As in the previous case, even values of *m* will generate a JC interaction, odd values will generate an anti-JC interaction. For values of *m* that are multiples of four, the light-matter interaction cancels out.

From Table 1 we see that the XY8 sequence achieves similar values for the effective coupling strength as the XXYY sequence. At the same time, however, it satisfies *γ*_*x*_ = *γ*_*y*_ = 0 and it is thus much more robust with respect to pulse imperfections.

As already pointed out above, some pulse sequences give rise to effective couplings of the form ${\tilde{H}}_{{\rm{int}}}^{{\rm{eff}}} \sim {\sigma }_{x}(a+{a}^{\dagger })$. This interaction plays a prominent role for various quantum control schemes^58,59^, but it also appears in the modeling of light-matter interactions in the so-called ultrastrong-coupling regime^60,61^, where the usual rotating-wave approximation is no longer applicable.

The simplest sequences giving rise to such a coupling are ${X}_{{t}_{1}}{X}_{{t}_{2}}$ for *m* = 0 and ${Y}_{{t}_{1}}{Y}_{{t}_{2}}$ for *m* ≠ 0. In both cases, the condition *γ*_*z*_ = 0 requires that *t*_2_ = *t*_1_ + *T*/2, and *t*_1_ is the only free parameter. For ${X}_{{t}_{1}}{X}_{{t}_{1}+T/2}$ and *m* = 0, the effective parameters are *η*_*y*_ = 0 and *η*_*x*_ = 1. The effective interaction then assumes the anticipated form,

28 $${\tilde{H}}_{{\rm{int}}}^{{\rm{eff}}}=\eta g\left({a}^{\dagger }{e}^{i\phi }+{\rm{H.c.}}\right){\sigma }_{x},$$

with *η* = 0.5 and *ϕ* = 0. The same is true for the ${Y}_{{t}_{1}}{Y}_{{t}_{1}+T/2}$ sequence and *m* odd, but with parameters

29 $$\eta =\frac{1}{\pi m}{\sin }^{2}(\pi m/2)$$

and *ϕ* = *πm*(2*t*_1_/*T* + 1) + *π*/2. Table 1c summarizes the values of *η* obtained for different *m* for the *XX* and the *YY* sequence.

To realize the full quantum Rabi model, we can choose a finite effective detuning *Δ*_eff_ and increase the rotation angle of each pulse to *π* + *δθ* with *δθ* ≪ *π*. In the case of the ${Y}_{{t}_{1}}{Y}_{{t}_{1}+T/2}$ sequence with *m* odd, the resulting effective Hamiltonian is then given by

30 $${\tilde{H}}_{{\rm{int}}}^{{\rm{eff}}}=\frac{\upsilon }{2}{\sigma }_{y}+\eta g\left({a}^{\dagger }{e}^{i\phi }{e}^{i{\Delta }_{{\rm{eff}}}t}+{\rm{H.c.}}\right){\sigma }_{x},$$

where *υ* = 2*δθ*/*T*. In the respective rotating frame, Eq. (30) describes the coupling of an effective TLS with frequency *ω*_0_ ≡ *υ* to a cavity mode of frequency *ω*_*c*_ ≡ *Δ*_eff_ and coupling strength *g*_eff_ = *ηg*. These parameters can be tuned independently through an appropriate choice of pulse parameters, enabling the system to reach the ultrastrong coupling regime, *g*_eff_ ≳ *ω*_0_, *ω*_*c*_, within this effective description.

**Fig. 3:**
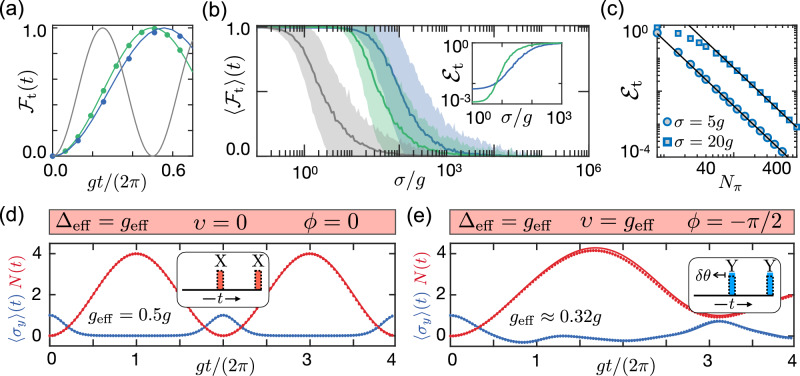
Protected Jaynes-Cummings and Rabi interactions. **a** Plot of the transfer fidelity as a function of time for the pulse sequences XY8_*m*=2_ (blue) and XXYY_*m*=0_ (green) in the absence of noise. The markers indicate the result of exact numerical simulations with an interpulse spacing *τ* = 0.1*g*^−1^, a pulse width *τ*_*π*_ = 0.1*τ* and *t*_1_ = *τ*/2, while solid lines follow an ideal evolution with the corresponding *g*_eff_. The shaded line represents the transfer fidelity for the bare JC-interaction. **b** For the same sequences the average transfer fidelity at time *T*_t_ = *π*/(2*g*_eff_) is plotted as a function of the noise strength *σ* and for a total of *N*_*π*_ = 64 pulses. While the solid lines represent the average values obtained as a result of 1000 independent noise realizations, randomly sampled from the probability distribution $P(\xi )={(2\pi {\sigma }^{2})}^{-1/2}\exp (-{\xi }^{2}/2{\sigma }^{2})$, the shaded area indicates its variation (one standard deviation). The inset shows the corresponding transfer error ${{\mathcal{E}}}_{{\rm{t}}}$ on a logarithmic scale. **c** Dependence of the average transfer error on the number of pulses *N*_*π*_ for the XY8_*m*=2_ sequence with *σ* = 5*g* (round markers) and *σ* = 20*g* (square markers). The solid lines indicate the analytic prediction from Eq. (31) for the same parameters. **d** Time evolution of the observables 〈*σ*_*y*_〉 and *N* = 〈*a*^†^*a*〉 for the XX_*m*=0_ sequence with parameters *τ* = 0.1*g*^−1^, *t*_1_ = *τ*, *τ*_*π*_ = 10^−2^*τ*, and *Δ* = *g*_eff_. The markers indicate the result obtained from exact numerical simulations, while the solid lines follow the prediction of the effective model in Eq. (30) with *Δ*_eff_ = *g*_eff_, *υ* = 0, and *ϕ* = 0. **e** The same as in (**d**) but for the sequence YY_*m*=1_ with a cavity detuning, *Δ* = 2*π*/*T* + *g*_eff_ and pulse area *π* + *δθ*, where *δθ* = *g*_eff_*T*/2. This simulation is compared to the effective model in Eq. (30) with *Δ*_eff_ = *υ* = *g*_eff_ and *ϕ* = − *π*/2. The small mismatch with the effective model, most visible at the peak values of *N*(*t*), is reduced when using a smaller interpulse spacing *τ*. The insets in (**d**) and (**e**) illustrate the pulse sequence, where the area enclosed by the dotted lines corresponds to *π*. Notably, in (**e**), the pulse area exceeds *π* by a small amount *δθ*.

To illustrate the effectiveness of these DD strategies, Fig. 3 shows the results of numerical simulations of the dynamics of the full cavity QED system for a finite number of echo pulses and in the presence of static frequency shifts of varying strength *σ*. In Fig. 3a, we first compare the evolution of the original JC model with that of the effective JC model obtained using the XXYY_*m*=0_ and the XY8_*m*=2_ sequences in the absence of noise. In all three cases we assume a resonant (effective) coupling and we plot the transfer fidelity ${{\mathcal{F}}}_{{\rm{t}}}(t)$ as a figure of merit. We see that already for a pulse spacing of *τ* = 0.1*g*^−1^, the effective evolution agrees very well with the analytic predictions from above and the effective coupling parameters listed in Table 1.

Including the influence of quasi-static noise, Fig. 3b demonstrates the increased robustness obtained for both pulse sequences for a total of *N*_*π*_ = 64 *π*-pulses. Under the same conditions, the inset shows the residual transfer error, ${{\mathcal{E}}}_{{\rm{t}}}=1-\langle {{\mathcal{F}}}_{{\rm{t}}}(t={T}_{{\rm{t}}})\rangle$, where *T*_t_ = *π*/(2*g*_eff_) now refers to the adjusted transfer time. We see that the error is reduced to values below 10^−2^, even when the noise strength exceeds the bare coupling strength. However, for small noise we also observe a saturation of the error, which arises from the finite pulse interval *τ* and depends on the pulse sequence. As we discuss in more detail in Methods and in Supplementary Note 2, we can use second-order perturbation theory to account for those effects. Focusing on the XY8_*m*=2_ sequence, which is more robust with respect to pulse errors and a finite width of the pulses, we obtain the transfer error

31 $${{\mathcal{E}}}_{{\rm{t}}}\approx \left[{\left(\frac{2\sigma }{3\pi }\right)}^{2}+1.88{g}^{2}\right]{\tau }^{2},$$

where *τ* = *T*_t_/*N*_*π*_. This result shows that the pulse spacing *τ* must be short compared to both the inverse noise strength and the inverse bare coupling constant. Once this condition is achieved, the error decreases very rapidly with the number of pulses, ${{\mathcal{E}}}_{{\rm{t}}}\propto {N}_{\pi }^{-2}$. When *σ* ≫ *g*, the second term in Eq. (31) becomes negligible and the suppression of the error compared to the evolution without DD pulses [see Eq. (7)] is approximately given by

32 $$\frac{{{\mathcal{E}}}_{{\rm{t}}}{| }_{{\rm{DD}}}}{{{\mathcal{E}}}_{{\rm{t}}}{| }_{{\rm{noDD}}}}\approx {\left(\frac{2}{3\eta {N}_{\pi }}\right)}^{2}.$$

This scaling is very accurately reproduced by the exact numerical simulations shown in Fig. 3c.

Finally, in Fig. 3d, e we also illustrate the implementation of the Rabi model under two slightly different conditions. In the first plot we consider an XX_*m*=0_ sequence with a finite *Δ*_eff_ = *g*_eff_. In the second plot, we consider a YY_*m*=1_ sequence, also with *Δ*_eff_ = *g*_eff_, but with an additional rotation angle *δθ* = *g*_eff_*T*/2 > 0. As explained in previously, the latter corresponds to an effective level splitting of *υ* = *g*_eff_ for the TLS. In both cases we find an excellent agreement between the exact dynamics and the one predicted by the effective model. Note, however, that for the simulation of the quantum Rabi model we have assumed a pulse length of *τ*_*π*_ = 10^−2^*τ*, which is an order of magnitude shorter than for the simulations of the JC model. This is due to the fact that the XX and YY sequences are more sensitive to pulse-width effects than the XXYY and XY8 sequences.

### Protecting cavity-mediated spin–spin interactions

In many cavity QED experiments, the primary purpose of the cavity mode is to mediate coherent interactions between otherwise decoupled TLSs. To study such applications, we extend our model to a scenario with two TLSs that are coupled to the same cavity mode with coupling strengths *g*_*j*_, where *j* = 1, 2. Assuming identical bare transition frequencies, the resulting Hamiltonian reads

33 $$H(t)=\Delta {a}^{\dagger }a+\mathop{\sum }\limits_{j=1}^{2}\frac{{\xi }_{j}(t)}{2}{\sigma }_{j}^{z}+\mathop{\sum }\limits_{j=1}^{2}{g}_{j}({\sigma }_{j}^{+}a+{\sigma }_{j}^{-}{a}^{\dagger }),$$

where the *ξ*_*j*_ are independent noise processes. When ∣*Δ*∣ ≫ *g*_*j*_, *ξ*_*j*_, the coupling to the cavity mode can be treated in perturbation theory and we obtain the following effective Hamiltonian

34 $$H(t)\approx \mathop{\sum }\limits_{j=1}^{2}\frac{\hat{{\xi }_{j}(t)}}{2}{\sigma }_{j}^{z}-J({\sigma }_{1}^{+}{\sigma }_{2}^{-}+{\sigma }_{1}^{-}{\sigma }_{2}^{+}),$$

where ${\hat{\xi }}_{j}(t)={\xi }_{j}(t)-\frac{| {g}_{j}{| }^{2}}{\Delta }(2{a}^{\dagger }a+1)$ and *J* = *g*_1_*g*_2_/*Δ*. In this detuned limit, the second term in Eq. (34) exchanges excitations between the TLSs, while only virtually populating the cavity mode. Specifically, after a time *T*_e_ = *π*/(4*J*), this term transforms the product state $\left\vert eg\right\rangle$ into the maximally entangled state $\left\vert \Psi \right\rangle =\frac{1}{\sqrt{2}}(\left\vert eg\right\rangle +i\left\vert ge\right\rangle )$, which is relevant for many quantum information processing applications. In the following we focus on this specific entanglement operation and use the entanglement fidelity

35 $${{\mathcal{F}}}_{{\rm{e}}}={\rm{Tr}}\{| \Psi \rangle \langle \Psi | \rho ({T}_{{\rm{e}}})\},$$

where *ρ*(*t*) is the full density operator of the cavity QED system, to quantify the accuracy of cavity-mediated interactions.

**Fig. 4:**
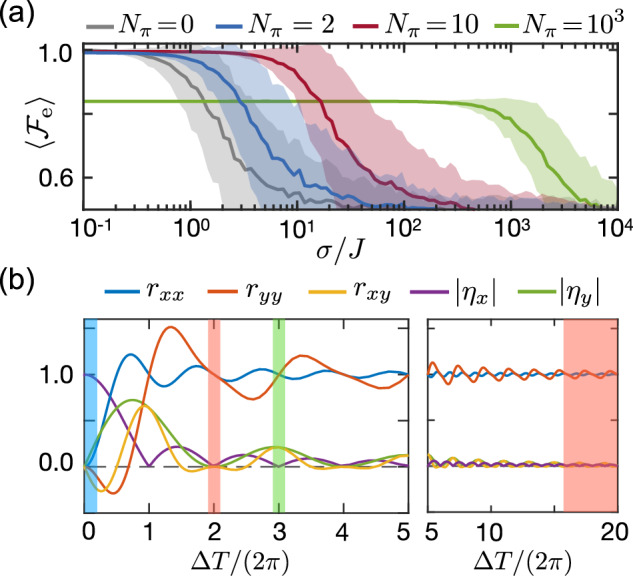
Entanglement fidelity and effective parameters. **a** Plot of the average entanglement fidelity $\langle {{\mathcal{F}}}_{{\rm{e}}}\rangle$ as a function of the noise strength *σ* for a cavity QED system with two TLSs. The TLSs are detuned by *Δ* = 30*g* and undergo cavity-mediated flip-flop interactions with strength *J* = *g*^2^/*Δ*. The solid lines (shaded areas) represent the average values (standard deviations) obtained from an average over 500 noise realizations for different numbers of *N*_*π*_ instantaneous *π*-pulses. **b** Dependence of the effective interaction parameters on *Δ* for the *X*_*T*/2_*X*_*T*_ pulse sequence. The red, blue and green shaded stripes indicate the regimes leading to flip-flop, Ising and squeezing interactions, respectively. See main text and Fig. 5 for more details.

Similar to the quantum state transfer analyzed in the previous section, the quality of the achieved spin-spin entanglement will decrease in the presence of uncontrolled fluctuations, *ξ*_*j*_(*t*). In Fig. 4a the gray line shows the average fidelity $\langle {{\mathcal{F}}}_{{\rm{e}}}\rangle$ as a function of the noise strength *σ*. For concreteness, we have assumed *g*_1_ = *g*_2_ = *g*, *Δ* = 30*g*, a cavity in the ground state, and independent, static fluctuations with 〈*ξ*_*i*_*ξ*_*j*_〉 = *σ*^2^*δ*_*ij*_. In this static limit, the noise-averaged fidelity decays as

36 $$\langle {{\mathcal{F}}}_{{\rm{e}}}\rangle \approx 1-\frac{1}{8}{\left(\frac{\sigma }{J}\right)}^{2},$$

and, therefore, the noise must be weak compared to the effective coupling *J* in order generate significant entanglement. Note that for a thermally populated cavity mode, the fluctuating AC Stark shift ~ *a*^†^*a* induces an additional random frequency shift with a strength *σ*_th_ = *g*^2^*n*_th_/∣*Δ*∣, where *n*_th_ is the thermal equilibrium occupation number of the mode^40^. However, to simplify the following discussion, we assume *n*_th_ = 0 and do not take this term explicitly into account.

In contrast to the resonant JC coupling discussed in Section “The noisy JC model", the effective Hamiltonian in Eq. (34) is already consistent with DD techniques. Specifically, the application of fast *π*-rotations on both TLSs simultaneously does not take the combined state out of the subspace of interest, $\{\left\vert eg\right\rangle ,\left\vert ge\right\rangle \}$, and is compatible with the discussed entanglement generation protocol. This can be understood by rewriting Hamiltonian (34) in the toggling frame. According to Eq. (12), in this frame the flip-flop term remains invariant, ${\tilde{\sigma }}_{1}^{+}(t){\tilde{\sigma }}_{2}^{-}(t)+{\rm{H.c.}}={\sigma }_{1}^{+}{\sigma }_{2}^{-}+{\rm{H.c.}}$, as long as the *π*-rotations are applied to both TLSs simultaneously. The resulting Hamiltonian is then given by

37 $$\tilde{H}(t)\approx \mathop{\sum }\limits_{j=1}^{2}\frac{\hat{{\xi }_{j}(t)}}{2}{f}_{z}(t){\sigma }_{j}^{z}-J({\sigma }_{1}^{+}{\sigma }_{2}^{-}+{\sigma }_{1}^{-}{\sigma }_{2}^{+}),$$

and for any pulse sequence with *γ*_*z*_ = 0, the static noise can be canceled to first order without affecting the interaction term.

In Fig. 4a, the blue line shows the resulting average fidelity $\langle {{\mathcal{F}}}_{{\rm{e}}}\rangle$ after introducing only a single *π*-rotation (on both TLSs) in the middle and at the end of the evolution. As expected, we see that the performance of the echoed case is significantly better than without the decoupling pulses. Note, however, that operation is still affected by noise, despite considering purely static fluctuations, *ξ*_*j*_(*t*) = *ξ*_*j*_, and ideal *π*-pulses. This is in stark contrast to more commonly investigated spin systems with Ising interactions of the form $J{\sigma }_{1}^{z}{\sigma }_{2}^{z}$^62^, where a single *π*-pulse applied to each TLS would be sufficient to cancel any static frequency shifts exactly. The difference in the current cavity setup arises from the fact that the noise and the interaction term in Eq. (37) do not commute and thus the overall dynamics is more involved.

This observation suggests that in order to improve cavity-mediated interactions, the interpulse spacing must satisfy not only *τ* ≲ *τ*_*c*_, but also *τ* ≲ 1/*σ*. Indeed, in Fig. 4a, the red line shows the fidelity for an evolution interrupted by a total of ten *X*-rotations of each TLS. In this case, the interpulse spacing *τ* is ten times shorter than in the previous example (blue line), increasing the level of tolerable noise by an order of magnitude.

However, the behavior discussed so far is valid only when the period *T* is long compared to *Δ*^−1^, i.e. *ΔT* ≫ 1. In this case, it is sufficient to apply the toggling-frame transformation to Hamiltonian (34) instead of the original JC Hamiltonian in Eq. (33). For example, in the sequences with up to ten pulses discussed above, we have *ΔT*/(2*π*) ≳ 22. In contrast, the green line in Fig. 4a shows a sequence with a total of 1000 pulses applied to each TLS, in which case *ΔT*/(2*π*) ≈ 0.22. In this regime, the effect of the noise is suppressed up to very high levels, but already for very low noise amplitudes, the fidelity ${{\mathcal{F}}}_{{\rm{e}}}$ is significantly degraded. We conclude that while the application of more and more pulses makes the evolution more robust, it also changes the form of the effective interaction, and the system can no longer be modeled by the simple flip-flop interaction as given in Eq. (37).

To model cavity-mediated interactions in a regime where the period *T* is comparable or shorter to *Δ*^−1^, the transformation to the toggling frame must be applied to the original Hamiltonian in Eq. (33), as it was done in Eq. (9) for a single TLS. Only after this transformation, we can use a second-order Magnus expansion to derive the time-averaged Hamiltonian (see Methods),

38 $${\tilde{H}}_{{\rm{eff}}}(nT)={\tilde{H}}_{{\rm{eff}}}^{(1)}(nT)+{\tilde{H}}_{{\rm{eff}}}^{(2)}(nT),$$

which describes the effective evolution of the whole system during the *n*-th time interval {*nT*, (*n* + 1)*T*}. Here, the first-order and second-order effective interactions are given by

39 $${\tilde{H}}_{{\rm{eff}}}^{(1)}(nT)=\frac{1}{T}\mathop{\int}\nolimits_{nT}^{(n+1)T}ds\tilde{H(s)}$$

and

40 $${\tilde{H}}_{{\rm{eff}}}^{(2)}(nT)=\frac{-i}{2T}\mathop{\int}\nolimits_{nT}^{(n+1)T}dt\mathop{\int}\nolimits_{nT}^{t}ds\,[\tilde{H(t)},\tilde{H(s)}],$$

respectively^55^. For convenience, we reorganize Eq. (38) as

41 $${\tilde{H}}_{{\rm{eff}}}(nT)={\tilde{H}}_{{\rm{sc}}}^{(1)}(nT)+{\tilde{H}}_{{\rm{ss}}}^{(2)}+{\tilde{H}}_{{\rm{corr}}}(nT),$$

where the first two terms represent the dominating effective interactions, while the third term contains additional unwanted contributions from the noise and other imperfections. These corrections will be discussed in more detail in Section “Imperfections” below and in Supplementary Note 2.

With the same conventions as introduced in the previous section for a single TLS, we obtain a spin-cavity coupling

42 $${\tilde{H}}_{{\rm{sc}}}^{(1)}(t)=\mathop{\sum }\limits_{j=1}^{2}\frac{{g}_{j}}{2}\left[({\eta }_{x}{\sigma }_{j}^{x}-i{\eta }_{y}{\sigma }_{j}^{y}){a}^{\dagger }{e}^{i{\Delta }_{{\rm{eff}}}t}+{\rm{H.c.}}\right]$$

and a second-order spin-spin interaction of the form

43 $${\tilde{H}}_{{\rm{ss}}}^{(2)}=-\frac{J}{2}\sum\limits_{\{u,v\}=\{x,y\}}{r}_{uv}\,{\sigma }_{1}^{u}{\sigma }_{2}^{v}.$$

Here, *J* = *g*_1_*g*_2_/*Δ* and we have introduced the additional dimensionless coefficients

44 $${r}_{uv}=-\frac{\Delta }{T}{\rm{Im}}\left[\mathop{\int}\nolimits_{0}^{T}dt\mathop{\int}\nolimits_{0}^{t}ds\,{\tilde{f}}_{u}^{* }(t){\tilde{f}}_{v}(s){e}^{-i\Delta (t-s)}\right],$$

where ${\tilde{f}}_{x}(t)={f}_{x}(t)$, ${\tilde{f}}_{y}(t)=-i{f}_{y}(t)$. For illustration, we consider in Fig. 4c the simple pulse sequence *X*_*T*/2_*X*_*T*_ and plot the values of *η*_*x*,*y*_ and the coefficients *r*_*xx*_, *r*_*yy*_ and *r*_*xy*_ = *r*_*yx*_ as a function of the detuning *Δ*.

From the form of the two contributions of ${\tilde{H}}_{{\rm{eff}}}$ given in Eq. (42) and Eq. (43), we see that there are different ways to obtain effective interactions between the TLSs. First, one can engineer an appropriate first-order Hamiltonian ${\tilde{H}}_{{\rm{sc}}}^{(1)}$, with an effective detuning *Δ*_eff_ that is large compared to the effective coupling *g*_eff_ ~ *ηg*. Similar to an unperturbed cavity QED system, this will generate cavity-mediated interactions with a scaling $\sim {g}_{{\rm{eff}}}^{2}/{\Delta }_{{\rm{eff}}}$. In addition, one can directly make use of the second-order Hamiltonian ${\tilde{H}}_{{\rm{ss}}}^{(2)}$, which represents an additional independent contribution that scales as *g*^2^/*Δ*. This combination, together with the strong dependence of all the coefficients on the detuning and the chosen pulse sequence, offers a large flexibility for engineering cavity-mediated interactions that are at the same time protected against noise. In the following, we illustrate these possibilities in terms of a few basic examples for the *X*_*T*/2_*X*_*T*_ sequence.

We start with the implementation of the flip-flop interaction

45 $${\tilde{H}}_{{\rm{ss}}}^{(2)}\simeq -J({\sigma }_{1}^{+}{\sigma }_{2}^{-}+{\sigma }_{1}^{-}{\sigma }_{2}^{+}).$$

To obtain this form, we require *r*_*xx*,*yy*_ → 1 and *r*_*xy*_, ∣*η*_*x*,*y*_∣ → 0. As we can see from Fig. 4c, this is always satisfied in the large detuning limit, *ΔT* ≫ 1. However, we can also identify specific conditions, *Δ* = *m* × 2*π*/*T* with *m* = 2, 4, . . . , where *r*_*xx*,*yy*_ = 1 while *r*_*xy*_ and *η*_*x*,*y*_ vanish. Thus, in these cases, the effective flip-flop interaction can be obtained already for small and moderate detunings or, equivalently, for short interpulse spacings. Note that this feature is not unique to the *X*_*T*/2_*X*_*T*_ sequence and similar conditions can also found for more complicated pulse sequences.

Another relevant type of coupling is the Ising interaction $\sim {\sigma }_{1}^{x}{\sigma }_{2}^{x}$. As we can see from Fig. 4c, there is no value for the detuning *Δ* for which only *r*_*xx*_ is non-vanishing. Therefore, in this case, we apply a different approach and set *Δ* = *Δ*_eff_, with *Δ*_eff_*T* ≪ 1. In this limit the first-order coupling dominates and the effective Hamiltonian is

46 $${\tilde{H}}_{{\rm{sc}}}^{(1)}(t)\simeq {g}_{{\rm{eff}}}({a}^{\dagger }{e}^{i{\phi }_{x}}{e}^{i{\Delta }_{{\rm{eff}}}t}+{\rm{H.c.}}){S}_{x}$$

with *g*_eff_ = ∣*η*_*x*_∣*g*/2 and ${S}_{x}={\sum }_{j}({g}_{j}/g){\sigma }_{j}^{x}$. If, in addition to *Δ*_eff_*T* ≪ 1, we choose *g*_eff_ ≪ *Δ*_eff_, the effect of ${\tilde{H}}_{{\rm{sc}}}^{(1)}$ is well described by the spin-spin Hamiltonian

47 $${\tilde{H}}_{{\rm{ss}}}^{(1)}\simeq -\frac{{g}_{{\rm{eff}}}^{2}}{{\Delta }_{{\rm{eff}}}}{S}_{x}^{2},$$

which can be derived from Eq. (46) using second-order perturbation theory.

Finally, another relevant application is the implementation of squeezing interactions $\sim ({\sigma }_{1}^{+}{\sigma }_{2}^{+}+{\sigma }_{1}^{-}{\sigma }_{2}^{-})$. To do so we combine the two strategies from above and fix the detuning as *Δ* = 2*πm*/*T* + *Δ*_eff_, with *m* = 3 and *Δ*_eff_ ≪ *Δ*. In this regime, the second-order Hamiltonian ${\tilde{H}}_{{\rm{ss}}}^{(2)}$ follows a flip-flop interaction, while the first-order term ${\tilde{H}}_{{\rm{sc}}}^{(1)}$ contributes the effective Ising interaction ${\tilde{H}}_{{\rm{ss}}}^{(1)}\simeq -{g}_{{\rm{eff}}}^{2}/{\Delta }_{{\rm{eff}}}{S}_{y}^{2}$ with *g*_eff_ = ∣*η*_*y*_∣*g*/2. If *Δ*_eff_ is chosen to be *Δ*_eff_ = −*πm*∣*η*_*y*_∣^2^/*T*, the combination of these two distinct interactions results in

48 $${\tilde{H}}_{{\rm{ss}}}\simeq \tilde{J}{\sigma }_{1}^{+}{\sigma }_{2}^{+}+{\tilde{J}}^{* }{\sigma }_{1}^{-}{\sigma }_{2}^{-}$$

with $\tilde{J}=-J(1-i| {\eta }_{y}| )$. When generalized to multiple TLSs, this Hamiltonian is equivalent to a two-axis squeezing Hamiltonian ${S}_{x}^{2}-{S}_{y}^{2}$, as relevant for quantum sensing applications^63^. This squeezing interaction will directly benefit from the dynamical protections, as we will discuss in the next section.

**Fig. 5:**
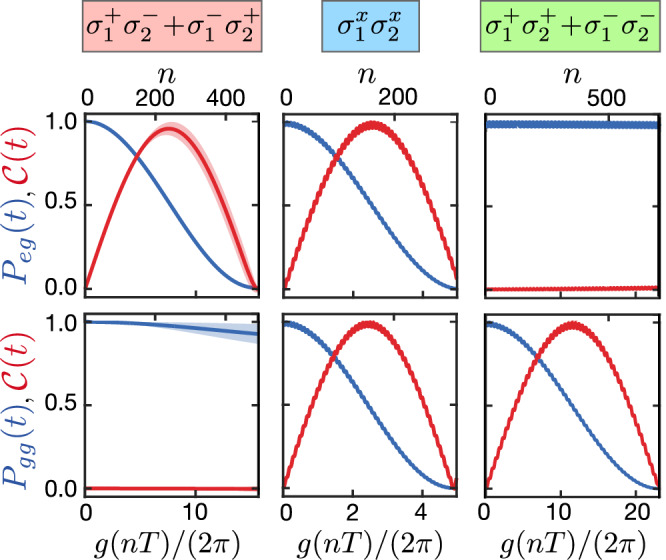
Numerical benchmarking of the different types of effective flip-flop (left), Ising (middle) and squeezing (right) interactions discussed in the main text. The upper (lower) panels show the exact time evolution of initial state $\left\vert eg\right\rangle$ ($\left\vert gg\right\rangle$) in terms of the population *P*_*eg*_ (*P*_*gg*_) in blue and the concurrence ${\mathcal{C}}$ of the reduced TLSs state in red. The solid lines and shaded areas represent the mean values and standard deviation of these quantities, as obtained from averaging over 500 realizations of static fluctuations with strength *σ* = 0.3*g*. In all simulations we assume an *X*_*T*/2_*X*_*T*_ sequence with a pulse duration of *τ*_*π*_ = 0.01*τ*. The other relevant parameters for the left panel are *m* = 2 and *T* = 0.2*g*^−1^. For the Ising and the squeezing interactions these are *m* = 0, *T* = 0.1*g*^−1^ and *Δ*_eff_ = 10*g*, and *m* = 3, *T* = 0.2*g*^−1^, and *Δ*_eff_ = − 2.16*g*, respectively. The relevant detunings *Δ* are indicated by the respective colored bars in Fig. 4b.

In Fig. 5, we benchmark the three different types of spin-spin interactions discussed above by exact numerical simulations of Hamiltonian (33), interrupted by finite-width *π*-pulses of duration *τ*_*π*_ = 10^−2^*τ*. For concreteness, we consider the time evolution of two distinct initial states, $\left\vert eg\right\rangle$ and $\left\vert gg\right\rangle$, and plot the resulting probabilities *P*_*eg*_(*t*) and *P*_*gg*_(*t*) together with the concurrence ${\mathcal{C}}(t)$ of the reduced state of the two TLSs as a function of time. Each of the columns shows the results of one type of spin-spin interaction, with the parameters of the XX sequence adjusted accordingly (see details in the figure caption). In all cases, we observe an excellent agreement between the full dynamics and the effective model, even under conditions where the noise strength is comparable to the bare coupling strength *g* and therefore exceeds by far the effective interaction strength, *σ* ≫ *J*. Note, that in these examples we have assumed a rather large number of pulses, as indicated by the number of periodic repetitions, *n* = *N*_*π*_/2. This could be relaxed, for example, in the case of flip–flop interactions, by choosing higher values of *m* to increase the period *T* while keeping *Δ* (and thus *J*) constant.

Finally, let us emphasize that the different interaction engineering strategies presented here are not exhaustive and can be combined, interchanged and optimized, depending on the application. For example, by using a XXYY sequence, the flip–flop interaction can also be realized via a detuning first-order effective JC interaction with a coupling strength ${J}_{{\rm{eff}}}\approx {g}_{{\rm{eff}}}^{2}/{\Delta }_{{\rm{eff}}}$, which can be significantly larger than the second-order coupling *J* assumed in the example above.

### Imperfections

After having explored various different ways to employ pulsed DD schemes for engineering protected cavity QED Hamiltonians, let us now take a closer look at the potential gain one can achieve with this strategy under realistic conditions. To do so, we must, in addition to frequency fluctuations and pulse imperfections, also include the Markovian decay of the cavity mode with rate *κ*, which we have omitted so far from our analysis.

As a first step, however, we address purely coherent errors, which arise, for example, from nonideal pulses or from an incomplete suppression of noise. To treat such errors in a systematic manner, we evaluate the correction Hamiltonian *H*_corr_ introduced in Eq. (41) up to second order in the coupling *g* and write the result as

49 $$\begin{array}{lll}{\tilde{H}}_{{\rm{corr}}}\,\simeq \,{\tilde{H}}_{{\rm{corr}}}^{(0)}+\sum\limits_{j}({\xi }_{j}T)\,{\tilde{H}}_{{\rm{corr}}}^{(1)}\\\qquad \,+\,\sum\limits_{j}{({\xi }_{j}T)}^{2}\,{\tilde{H}}_{{\rm{corr}}}^{(2,1)}+({\xi }_{1}{\xi }_{2}{T}^{2})\,{\tilde{H}}_{{\rm{corr}}}^{(2,2)}.\end{array}$$

The full expressions for each of these contributions are presented together with the derivation of all the results in this section in Methods.

The first term in Eq. (49) is independent of the noise and represents, for example, residual errors arising from a finite width or spacing between the pulses. From an explicit evaluation of this term for XXYY and XY8 sequences, we find that the main contribution arises from a nonvanishing spacing between the pulses ~ *gT* and results in an effective Hamiltonian of the form

50 $${H}_{{\rm{corr}}}^{(0)}\approx \frac{{g}^{2}T}{4}{G}_{2,z}^{(0)}\left({a}^{\dagger 2}+{a}^{2}\right){\sigma }_{z}.$$

The numerical prefactor, which is ${G}_{2,z}^{(0)}=0.25$ for XXYY_*m*=0_ and ${G}_{2,z}^{(0)}\approx 0.20$ for XY8_*m*=2_, shows no strong dependence on the applied sequence. For the latter case, the resulting transfer error is given by the second term in Eq. (31). Due to a larger effective coupling strength and shorter period, an even lower error can be achieved with the XXYY_*m*=0_ sequence, as confirmed by the inset of Fig. 3b for *σ* → 0.

**Fig. 6:**
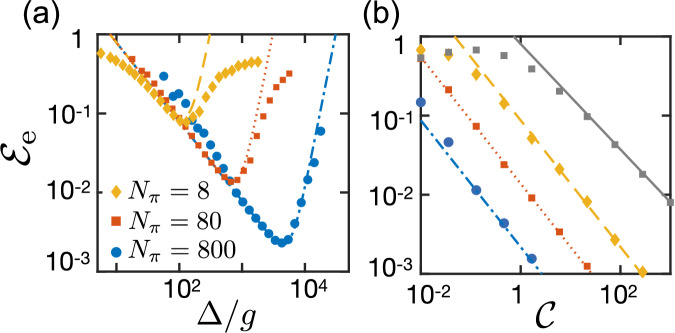
Entanglement error and cooperativity. **a** Plot of the entanglement error ${{\mathcal{E}}}_{{\rm{e}}}=1-\langle {{\mathcal{F}}}_{{\rm{e}}}\rangle$ as a function of the detuning *Δ* and for different numbers of pulses *N*_*π*_. For this simulation, we consider the implementation of a flip-flop interaction with an XY8 sequence (*t*_1_ = 0.5*τ* and *τ*_*π*_ = 0.01*τ*) and for *κ* = 10*g* and *σ* = 0.1*g* (${\mathcal{C}}=1$). The averages are taken over 500 realizations of static fluctuations. The diamond, square and round markers are the result of numerical simulations, and the dashed, dotted and dashed-dotted lines represent the prediction of Eq. (62) for *N*_*π*_ = 8, 80 and 800, respectively. Note that we limit the possible values of the detuning to $\Delta =g\sqrt{m{N}_{\pi }}$ with *m* = 4, 8, 12. **b** The minimum achievable error ${{\mathcal{E}}}_{\min }$ is plotted versus the cooperativity ${\mathcal{C}}$ for different number of pulses *N*_*π*_. The markers represent the results of numerical simulations with *Δ* ≈ *Δ*_opt_, while the lines indicate the scaling given in Eq. (63). The gray square markers and the solid line represent exact results and the scaling in Eq. (60) for the pulse-free case.

Note that in the absence of noise and up to the order considered in the expansion, a finite width of pulses *τ*_*π*_ has no direct influence on the fidelity. However, this advantage is lost when larger values of *σ* are taken into account, where we find that XXYY_*m*=0_ with *τ*_*π*_ > 0 performs worse than the corresponding XY8_*m*=2_ sequence. For the implementation of off-resonant spin-spin interactions, *τ*_*π*_ must be short compared to *σ*^−1^ and *g*^−1^, but the pulses can be slow compared to *Δ*^−1^. For example, this is the case for most of the results presented in Fig. 6. Thus, even in this limit, the implementation of the decoupling scheme remains experimentally feasible.

The second term in Eq. (49) captures first-order corrections from the noise. For a resonant JC interaction, its main effect is a random modulation of the effective coupling strength,

51 $${g}_{{\rm{eff}}}(\xi )\simeq g\left(\eta +\xi T{O}_{1}\right),$$

where *O*_1_ is a sequence-dependent numerical factor. On average, this correction induces a transfer error ~ (*στ*)^2^, which, for the XY8_*m*=2_ sequence, is captured by the first term in Eq. (31). For the XXYY_*m*=0_ sequence, we find that *O*_1_ = 0 and this contribution vanishes. Therefore, we must evaluate the higher-order corrections in the second line of Eq. (49), which scale as ~ *ξ*^2^. These terms result in a transfer error of

52 $${{\mathcal{E}}}_{{\rm{t}}}\approx 2.61\times \frac{{(2\sigma )}^{4}{\tau }^{2}}{{\eta }^{2}{g}^{2}}{{\Gamma }_{{\rm{pw}}}}^{2},$$

which now scales as *σ*^4^. For the XXYY_*m*=0_ sequence with *τ*_*π*_/*τ* = 0.1 the numerical factor is *Γ*_pw_ ≈ 0.4, but it vanishes for *τ*_*π*_ = 0. In this limit, the error is determined by even high-order processes, which are no longer included in our error model.

For the realization of cavity-mediated spin-spin interactions, we can choose *m* = 4, 8, 12 such that also for the XY8_*m*_ sequence, the first-order coupling correction vanishes, *O*_1_ = 0. The main correction then arises from a modulation of the exchange coupling term, *J* → *J* + *δJ*, where

53 $$\delta J\approx -\frac{J{T}^{2}}{{4}^{3}{5}^{2}}{({\xi }_{1}-{\xi }_{2})}^{2}.$$

Interestingly, also in this case the lowest-order correction is quadratic in the *ξ*_*i*_, which for uncorrelated noise leads to an entanglement error of

54 $${{\mathcal{E}}}_{{\rm{e}}}\approx \frac{12{\pi }^{2}}{{5}^{4}}{(\sigma \tau )}^{4}.$$

To account for incoherent losses of the cavity mode, the system must be described by a master equation of the form

55 $$\dot{\rho }=-i[H(t),\rho ]+\frac{\kappa }{2}\left(2a\rho {a}^{\dagger }-{a}^{\dagger }a\rho -\rho {a}^{\dagger }a\right),$$

where *ρ* is the density operator of the full system and *κ* is the cavity decay rate.

In the case of resonant interactions, we are usually interested in the regime, *g*_eff_ ≫ *κ*, where a coherent exchange of excitations between the TLS and the cavity can take place. This condition implies *κT* ≪ 1 and we can simply replace *H*(*t*) by ${\tilde{H}}_{{\rm{eff}}}^{(1)}(t)$ in Eq. (55) to evaluate the combined system dynamics with a lossy cavity. The DD pulses then suppress the effect of the noise approximately as *σ* → *σ*/*N*_*π*_, while having no influence on the Markovian decay of the cavity mode. Thus, we obtain the relaxed strong-coupling condition,

56 $${g}_{{\rm{eff}}} > \sigma /{N}_{\pi },\kappa ,$$

as long as a sufficiently large number of pulses is applied.

In the weak-coupling or far-detuned regime, the cavity is only virtually populated and its dynamics can be adiabatically eliminated. For the bare JC model, this procedure predicts a decay of the excited state of the TLS with rate *γ*_0_ ≃ *g*^2^*κ*/(*Δ*^2^ + *κ*^2^/4). In Supplementary Note 3 we extend this analysis and derive an equivalent effective rate for arbitrary DD pulses. In the limit of large detuning *Δ* ≫ *T*^−1^, *κ* the resulting decay rates for the ground and excited state simplify to

57 $${\gamma }_{{\rm{eff}}}^{e}\simeq {\gamma }_{{\rm{eff}}}^{g}\simeq \frac{{g}^{2}}{2{\Delta }^{2}}\kappa .$$

This is approximately half of *γ*_0_, the rate of the excited state in an undriven TLS. However, in the modulated system, both states are affected equally and the resulting decoherence rate of a superposition state remains *γ*_0_.

In the opposite limit *ΔT* ~ 1, and for sequences with a nonvanishing first-order JC-like coupling, *η* ≠ 0, our analysis predicts the decay of a TLS initialized in state $\left\vert e\right\rangle$ with a rate

58 $${\gamma }_{{\rm{eff}}}^{e}\simeq \frac{{g}_{{\rm{eff}}}^{2}\kappa }{{\Delta }_{{\rm{eff}}}^{2}+{\kappa }^{2}/4}.$$

This rate is as expected for a JC-model with effective parameters, *g*_eff_, *Δ*_eff_, and *κ*. Note, however, that also in this regime the ground state decays with a small, but nonvanishing rate ${\gamma }_{{\rm{eff}}}^{g}\approx {\gamma }_{0}$.

For general detunings, the expressions for ${\gamma }_{{\rm{eff}}}^{e/g}$ are more involved, but can be evaluated for arbitrary pulse sequences, as described in Supplementary Note 3. A typical behavior for the XY8 sequence is shown in Supplementary Fig. 1. Overall, we find that away from individual resonances the simple scaling ${\gamma }_{{\rm{eff}}}^{e/g}\approx {\gamma }_{0}$ provides a useful general estimate for the effective decay rates.

Finally, let us return to the application of generating entanglement via a cavity-mediated flip-flop interaction in the far-detuned regime. For the bare JC model, but taking both frequency noise and cavity decay into account, the resulting entanglement fidelity is approximately given by

59 $$\langle {{\mathcal{F}}}_{{\rm{e}}}\rangle \approx 1-\frac{4}{{\pi }^{2}}{\left(\frac{{T}_{{\rm{e}}}}{{T}_{2}^{* }}\right)}^{2}-{\gamma }_{0}{T}_{{\rm{e}}}$$

with ${T}_{2}^{* }=\sqrt{2}/\sigma$. This expression reaches a maximum for ${\Delta }_{{\rm{opt}}}={(\pi {g}^{4}\kappa {({T}_{2}^{* })}^{2}/2)}^{1/3}$, where

60 $$\langle {{\mathcal{F}}}_{{\rm{e}}}\rangle \approx 1-\frac{3}{8}{\left(\frac{\pi }{{\mathcal{C}}}\right)}^{2/3}.$$

Therefore, in this detuned regime, the maximal fidelity depends only on a single parameter, namely the cooperativity

61 $${\mathcal{C}}=\frac{{g}^{2}}{\sigma \kappa }.$$

A similar scaling as in Eq. (60) is also found for several other applications in cavity QED, where larger values of *κ* can be compensated by correspondingly lower dephasing rates.

In Fig. 6, we simulate the same entangling gate under the influence of DD pulses. Specifically, for these simulations we consider the XY8_*m*_ sequence, which is very robust with respect to pulse imperfections and thus well-suited for implementing DD schemes with a very large number of pulses^26^. For detunings *Δ* = *m* × 2*π*/*T* with *m* = 4, 8, . . . the first-order coupling vanishes, *g*_eff_ = 0, and the effective system evolution is well-described by the flip-flop Hamiltonian in Eq. (45) with *J* = *g*_1_*g*_2_/*Δ*. To ensure decoupling during all periods, we choose the final time *t* ≃ *T*_e_ as a multiple of the period *T*, and thus set $\Delta =g\sqrt{m{N}_{\pi }}$. For a fixed number of pulses *N*_*π*_, we vary *Δ* by changing the value of *m* = 4, 8, 12,... .

Under these conditions, our error analysis from above predicts an average entangling fidelity of

62 $$\langle {{\mathcal{F}}}_{{\rm{e}}}\rangle \approx 1-\frac{3{\pi }^{2}}{{5}^{4}{N}_{\pi }^{4}}{\left(\frac{{T}_{{\rm{e}}}}{{T}_{2}^{* }}\right)}^{4}-{\gamma }_{0}{T}_{{\rm{e}}},$$

where, as discussed above, we can use *γ*_0_ = *g*^2^*κ*/*Δ*^2^ as the approximate decay rate for large detunings. This expression shows that the DD pulses not only increase the coherence time, but also change the scaling of the error for static noise. Therefore, the fidelity is optimized at a different detuning ${\Delta }_{{\rm{opt}}}=2.13\,{({g}^{8}{N}_{\pi }^{4}\kappa /{\sigma }^{4})}^{1/5}$, where it reaches a maximal value of

63 $$\langle {{\mathcal{F}}}_{{\rm{e}}}\rangle \approx 1-0.46\,{\left(\frac{1}{{N}_{\pi }{\mathcal{C}}}\right)}^{4/5},$$

predicting an almost linear gain with the number of applied *π*-pulses.

To confirm our analytical estimates, in Fig. 6a, we show the excellent correspondence between Eq. (62) and the entanglement fidelity obtained from exact numerical simulation over a wide range of parameters, as long as overall error is small enough, ${{\mathcal{E}}}_{{\rm{e}}}\lesssim 0.1$. In Fig. 6b, we also compare the optimal fidelities given in Eq. (60) and Eq. (63) with the corresponding numerically optimized results for different values of the cooperativity ${\mathcal{C}}$. We vary the latter by changing *σ* while maintaining a fixed *κ* = 10*g*. Again, the exact results follow very accurately the predicted trends and confirm the boost of the effective cooperativity by several orders of magnitude. The same enhancement will also benefit many other applications, for example, cavity-mediated spin squeezing, where the minimal spin-squeezing parameter is predicted to scale as ${\xi }_{{\rm{squeez}}}^{2} \sim 1/\sqrt{{N}_{\pi }{\mathcal{C}}}$^64^.

Note that all analytical and numerical predictions in this work assume that any residual incoherent decay of the TLS with rate ${T}_{1}^{-1}$ is negligible on the timescales of interest. Such a Markovian decay is not affected by DD and will contribute a trivial error *O*(*T*_e_/*T*_1_) to any coherent operation.

## Discussion

In summary, we have proposed a general pulsed DD strategy for protecting cavity-QED systems against quasi-static frequency fluctuations. Our analysis revealed that this approach not only suppresses the effects of noise but also enables the engineering and modulation of different types of interactions by simply adjusting the pulse parameters. Furthermore, we provided a comprehensive analysis of the effective interactions and residual errors that arise for a given DD sequence, facilitating the optimization of this technique for specific experimental setups.

As a relevant application, we demonstrated how cavity-mediated entanglement operations can be systematically enhanced by increasing the number of applied *π*-rotations using the experimentally robust XY8 sequence. These findings are particularly relevant for solid-state cavity-QED experiments with spin qubits^9,18–21^ or rare-earth dopants^10–13^, where frequency inhomogeneities and slow frequency drifts present common experimental challenges. However, these techniques also offer a robust and versatile approach for engineering effective light-matter and spin–boson interactions in a wide range of other settings.

## Methods

### Derivation of the full effective Hamiltonian

In this note we summarize the details of the derivation of the full second-order Hamiltonian given in Eq. (38), which contains the targeted effective first-order and second-order interactions, as well as additional correction terms. For the derivation of this effective model we start from the cavity QED Hamiltonian in Eq. (33) in the toggling frame, where

64 $$\begin{array}{rcl}\tilde{H(t)}&=&\mathop{\sum }\limits_{j=1}^{2}\frac{{\xi }_{j}}{2}{\overrightarrow{f}}_{\!\!\xi }(t)\cdot {\overrightarrow{\sigma }}_{\!\!j}\\ &&+\mathop{\sum }\limits_{j=1}^{2}\frac{{g}_{j}}{2}({\overrightarrow{f}}_{{\rm{JC}}}(t)\cdot {\overrightarrow{\sigma }}_{\!\!j}\,{a}^{\dagger }{e}^{i\Delta t}+{\rm{H.c.}}).\end{array}$$

Note that in contrast to the analysis in the main text, here we are more general and include pulses of finite duration. In this case the modulation functions ${\overrightarrow{f}}_{\xi }(t)$ and ${\overrightarrow{f}}_{{\rm{JC}}}(t)$ are defined by

65 $$\begin{array}{rcl}{U}_{\pi }^{\dagger }(t){\sigma }_{j}^{z}{U}_{\pi }(t)&=&{\overrightarrow{f}}_{\!\!\xi }(t)\cdot {\overrightarrow{\sigma }}_{j},\\ {U}_{\pi }^{\dagger }(t){\sigma }_{j}^{-}{U}_{\pi }(t)&=&{\overrightarrow{f}}_{{\rm{JC}}}(t)\cdot {\overrightarrow{\sigma }}_{j}.\\ \end{array}$$

**Fig. 7:**
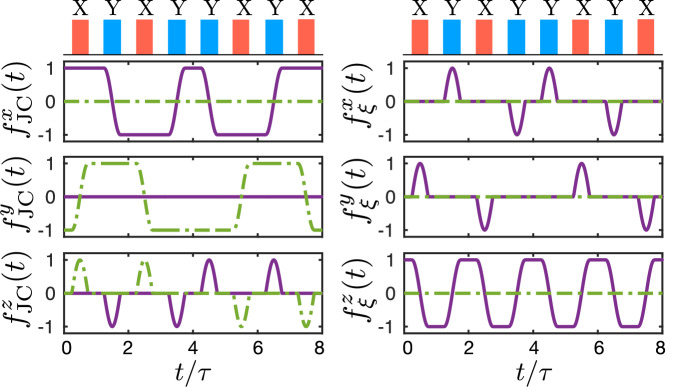
Illustration of the time-dependence of the modulation functions ${\overrightarrow{f}}_{{\!\!\rm{JC}}}(t)$ (left plots) and ${\overrightarrow{f}}_{\!\!\xi }(t)$ (right plots) for an XY8 sequence with pulses of width *τ*_*π*_ = 0.5*τ*. The solid and dashed lines indicate the real and imaginary parts, respectively. In the limit, *τ*_*π*_ → 0, the functions ${f}_{{\rm{JC}}}^{z}$ and ${f}_{{\rm{\xi }}}^{x,y}$ vanish identically.

Thus, in this general case, the transformation to the toggling frame mixes the different Pauli operators. For illustration, Fig. 7 shows the form of these functions for the XY8 sequence and for pulses with a deliberately long duration of *τ*_*π*_ = 0.5*τ*. For instantaneous pulses, these functions simplify to ${\overrightarrow{f}}_{\!\!{\rm{JC}}}(t)=({f}_{x}(t),-i{f}_{y}(t),0)$ and ${\overrightarrow{f}}_{\xi }(t)=(0,0,{f}_{z}(t))$, where the *f*_*k*_(*t*) behave according to Eq. (12).

In a second step, we proceed by implementing another unitary transformation into an interaction picture with respect to the noise term ${\tilde{H}}_{\xi }(t)={\sum }_{j}{\xi }_{j}{f}_{\xi }^{z}(t){\sigma }_{j}^{z}/2$. The purpose of this transformation is not immediately obvious, but, as we show below, it allows us to capture relevant corrections terms ~ *g*^2^*ξ*^2^, which we would not obtain from a direct second-order expansion. In this new frame, Eq. (64) transforms into

66 $$\begin{array}{lll}\tilde{H(t)}&=&\mathop{\sum }\limits_{j=1}^{2}\frac{{\xi }_{j}}{2}{\overrightarrow{F}}_{\!\!\xi ,j}(t)\cdot {\overrightarrow{\sigma }}_{j}\\ &&+\mathop{\sum }\limits_{j=1}^{2}\frac{{g}_{j}}{2}\left({\overrightarrow{F}}_{\!\!{\rm{JC,j}}}(t)\cdot {\overrightarrow{\sigma }}_{j}\,{a}^{\dagger }+{\rm{H.c.}}\right),\end{array}$$

where the updated modulation functions now depend on ${\varphi }_{j}(t)={\xi }_{j}\mathop{\int}\nolimits_{0}^{t}ds{f}_{z}^{\xi }(s)$ as

$$\begin{array}{rcl}{F}_{{\rm{JC}},j}^{x}(t)&=&[{f}_{{\rm{JC}},j}^{x}(t)\cos {\varphi }_{j}(t)+{f}_{{\rm{JC}},j}^{y}(t)\sin {\varphi }_{j}(t)]{e}^{i\Delta t},\\ {F}_{{\rm{JC}},j}^{y}(t)&=&[{f}_{{\rm{JC}},j}^{y}(t)\cos {\varphi }_{j}(t)-{f}_{{\rm{JC}},j}^{x}(t)\sin {\varphi }_{j}(t)]{e}^{i\Delta t},\\ {F}_{\xi ,j}^{x}(t)&=&{f}_{\xi ,j}^{x}(t)\cos {\varphi }_{j}(t)+{f}_{\xi ,j}^{y}(t)\sin {\varphi }_{j}(t),\\ {F}_{\xi ,j}^{y}(t)&=&{f}_{\xi ,j}^{y}(t)\cos {\varphi }_{j}(t)-{f}_{\xi ,j}^{x}(t)\sin {\varphi }_{j}(t),\end{array}$$

and ${F}_{{\rm{JC}},j}^{z}(t)={f}_{{\rm{JC}},j}^{z}(t)$, and ${F}_{\xi ,j}^{z}(t)=0$. Note that, in this frame, the first term in Eq. (66) accounts exclusively for effects related to a finite pulse width and vanishes for instantaneous pulses.

At this stage we perform a Magnus expansion for the evolution operator during the *n*-th time interval [*nT*, (*n* + 1)*T*]. More precisely, we write

67 $${\mathcal{T}}{e}^{-i\mathop{\int}\nolimits_{nT}^{(n+1)T}dt\tilde{H(t)}}={e}^{-i\mathop{\sum }\nolimits_{k = 1}^{\infty }{H}_{{\rm{eff}}}^{(k)}(nT)T},$$

where ${\mathcal{T}}$ denotes the time-ordered exponential. By truncating this expansion after the second-order and by making use of the identity $[\overrightarrow{a}\cdot {\overrightarrow{\sigma }}_{j},\overrightarrow{b}\cdot {\overrightarrow{\sigma }}_{k}]=2i{\delta }_{j,k}\,(\overrightarrow{a}\times \overrightarrow{b})\cdot {\overrightarrow{\sigma }}_{k}$, we finally obtain the effective Hamiltonian

68 $$\begin{array}{rcl}{\tilde{H}}_{{\rm{eff}}}(nT)&=&\sum\limits_{j}\frac{{\xi }_{j}}{2}{\overrightarrow{{\mathcal{W}}}}_{j}\cdot {\overrightarrow{\sigma }}_{j}\\ &&+\sum\limits_{j}\frac{{g}_{j}}{2}\left\{{\overrightarrow{{\mathcal{O}}}}_{j}{a}^{\dagger }{e}^{i{\Delta }_{{\rm{eff}}}nT}+{\rm{H.c.}}\right\}\cdot {\overrightarrow{\sigma }}_{j}\\ &&+\sum\limits_{j}\frac{{g}_{j}^{2}T}{4}\left\{{\overrightarrow{{\mathcal{G}}}}_{2,j}{({a}^{\dagger })}^{2}{e}^{i2{\Delta }_{{\rm{eff}}}nT}+{\rm{H.c.}}\right\}\cdot {\overrightarrow{\sigma }}_{j}\\ &&+\sum\limits_{j}\frac{{g}_{j}^{2}T}{4}(2{a}^{\dagger }a+1){\rm{Re}}\left({\overrightarrow{{\mathcal{G}}}}_{3,j}\right)\cdot {\overrightarrow{\sigma }}_{j}\\ &&+\sum\limits_{\{u,v\}=\{x,y,z\}}\frac{{g}_{1}{g}_{2}T}{2}{\rm{Im}}({{\mathcal{G}}}_{uv})\,{\sigma }_{1}^{u}{\sigma }_{2}^{v}.\end{array}$$

Here we introduced the sets of dimensionless parameters ${\overrightarrow{{\mathcal{W}}}}_{j}={\overrightarrow{\Gamma }}_{j}+\frac{{\xi }_{j}T}{2}{\overrightarrow{{\mathcal{G}}}}_{0,j}$, and ${\overrightarrow{{\mathcal{O}}}}_{j}={\overrightarrow{O}}_{j}+{\xi }_{j}T{\overrightarrow{{\mathcal{G}}}}_{1,j}$, which in turn are given in terms of the pulse-dependent integrals

69 $${\overrightarrow{\Gamma }}_{j}=\frac{1}{T}\mathop{\int}\nolimits_{0}^{T}dt\,{\overrightarrow{F}}_{\xi ,j}(t),$$

70 $${\overrightarrow{O}}_{j}=\frac{1}{T}\mathop{\int}\nolimits_{0}^{T}dt\,{\overrightarrow{F}}_{{\rm{JC}},j}(t),$$

and

71 $${\overrightarrow{{\mathcal{G}}}}_{0,j}=\frac{1}{{T}^{2}}\iint {\overrightarrow{F}}_{\xi ,j}(t)\times {\overrightarrow{F}}_{\xi ,j}(s),$$

72 $${\overrightarrow{{\mathcal{G}}}}_{1,j}=\frac{1}{{T}^{2}}\iint {\overrightarrow{F}}_{{\rm{JC}},j}(t)\times {\overrightarrow{F}}_{\xi ,j}(s),$$

73 $${\overrightarrow{{\mathcal{G}}}}_{2,j}=\frac{1}{{T}^{2}}\iint {\overrightarrow{F}}_{{\rm{JC}},j}(t)\times {\overrightarrow{F}}_{{\rm{JC}},j}(s),$$

74 $${\overrightarrow{{\mathcal{G}}}}_{3,j}=\frac{1}{{T}^{2}}\iint {\overrightarrow{F}}_{{\rm{JC}},j}^{* }(t)\times {\overrightarrow{F}}_{{\rm{JC}},j}(s),$$

75 $$\begin{array}{rcl}{{\mathcal{G}}}_{uv}&=&\frac{1}{2{T}^{2}}\iint \,[\hat{u}\cdot {\overrightarrow{F}}_{{\rm{JC}},1}^{* }(t)][\hat{v}\cdot {\overrightarrow{F}}_{{\rm{JC}},2}(s)],\\ &&+\frac{1}{2{T}^{2}}\iint \,[\hat{u}\cdot {\overrightarrow{F}}_{{\rm{JC}},2}^{* }(t)][\hat{v}\cdot {\overrightarrow{F}}_{{\rm{JC}},1}(s)],\end{array}$$

where $\iint \equiv \mathop{\int}\nolimits_{0}^{T}dt\mathop{\int}\nolimits_{0}^{t}ds$ for clarity. It is noteworthy that the numerical value of these integrals depends only on the form of the pulse sequence, and the dimensionless parameters *ΔT* and *ξ*_*j*_*T*.

In the form given in Eq. (68), the effective Hamiltonian *H*_eff_ contains all interaction terms up to order (*gT*)^2^, but in view of the interaction representation assumed in Eq. (66), it still contains arbitrary orders of *ξ*_*j*_. Therefore, in a final step we expand ${\overrightarrow{F}}_{\xi ,j}(t)$ and ${\overrightarrow{F}}_{{\rm{JC}},j}(t)$ up to second order in *ξ*_*j*_ (assuming *φ*_*j*_(*t*) ≪ 1), leading to the decomposition of the integrals ${\overrightarrow{\Gamma }}_{j},{\overrightarrow{O}}_{j}$ and ${\overrightarrow{G}}_{k,j}$ in the fashion

76 $${\overrightarrow{{\mathcal{G}}}}_{k,j}={\overrightarrow{{\mathcal{G}}}}_{k}^{(0)}+({\xi }_{j}T){\overrightarrow{{\mathcal{G}}}}_{k}^{(1)}-\frac{1}{2}{({\xi }_{j}T)}^{2}{\overrightarrow{{\mathcal{G}}}}_{k}^{(2)},$$

and the decomposition of ${{\mathcal{G}}}_{uv}$ as

77 $$\begin{array}{rcl}{{\mathcal{G}}}_{uv}&=&{{\mathcal{G}}}_{uv}^{(0)}+\frac{1}{2}({\xi }_{1}+{\xi }_{2})T{{\mathcal{G}}}_{uv}^{(1)}\\ &&-\frac{1}{4}({\xi }_{1}^{2}+{\xi }_{2}^{2}){T}^{2}{{\mathcal{G}}}_{uv}^{(2,1)}-\frac{1}{4}({\xi }_{1}{\xi }_{2}){T}^{2}{{\mathcal{G}}}_{uv}^{(2,2)}.\end{array}$$

Hamiltonian (68) can then be reorganized accordingly as

78 $$\begin{array}{rcl}{\tilde{H}}_{{\rm{eff}}}&\approx &{\tilde{H}}_{{\rm{sc}}}^{(1)}+{\tilde{H}}_{{\rm{ss}}}^{(2)}+{\tilde{H}}_{{\rm{corr}}}^{(0)}+\sum\limits_{j}({\xi }_{j}T)\,{\tilde{H}}_{{\rm{corr}}}^{(1)}\\ &&+\sum\limits_{j}{({\xi }_{j}T)}^{2}\,{\tilde{H}}_{{\rm{corr}}}^{(2,1)}+({\xi }_{1}{\xi }_{2}{T}^{2})\,{\tilde{H}}_{{\rm{corr}}}^{(2,2)},\end{array}$$

where ${\tilde{H}}_{{\rm{sc}}}^{(1)}$ and ${\tilde{H}}_{{\rm{ss}}}^{(2)}$ are the interaction terms given in Eqs. (42) and (43) in the main text with ${\eta }_{x}\equiv {O}_{x}^{(0)}$, ${\eta }_{y}\equiv i{O}_{y}^{(0)}$ and ${r}_{uv}\equiv -\Delta T{{\mathcal{G}}}_{uv}^{(0)}$. All the remaining terms represent noise-induced and other pulse-related corrections. Note that while avoiding a full 4th-order Magnus expansion, our derivation still accounts for relevant correction terms scaling as ~ *g*^2^*ξ*^2^. The numerical values for the most relevant coefficients are summarized in Supplementary Table I for the sequences XXYY_*m*=0_, XY8_*m*=2_, and *X*_*T*/2_*X*_3*T*/4_*Y*_3*T*/4_*Y*_*T*_^48^.

## Supplementary information

Supplementary Information

## Data Availability

Data is available from the corresponding authors upon reasonable request. The codes used in this study are available from the corresponding authors upon reasonable request.

## Code availability

The codes used in this study are available from the corresponding authors upon reasonable request.
