# Supplementary material for: Engineering protected cavity-QED interactions through pulsed dynamical decoupling
Source: npj Quantum Inf. 2025 Nov 21;11(1):197. doi: 10.1038/s41534-025-01143-5 (PMC12747883; doi:10.1038/s41534-025-01143-5)
Supplement: Supplementary file 1 — Supplementary Information [file 41534_2025_1143_MOESM1_ESM.pdf]

Supplementary Material: Engineering protected cavity-QED interactions through pulsed dynamical decoupling

Supplementary Note 1: Errors in the JC state transfer

For all our numerical simulations and analytic estimates, we consider the limit of a purely static noise, $\tau_c/\tau \to 0$. In this note, we quantify how small static shifts in the experimental parameters affect the JC state transfer discussed in Section "The noisy Jaynes-Cummings Model". We consider deviations from the ideal JC Hamiltonian $H = g_0(\sigma_+ a + \sigma_- a^\dagger)$, modeled by the Hamiltonian

$$H = \frac{\xi}{2}\sigma_z + (g\sigma_+ a + g^*\sigma_- a^\dagger), \tag{S1}$$

where $g = g_0(1+\epsilon)$. Here ξ accounts for frequency fluctuations of the TLS, and $\epsilon \in \mathbb{C}$ for deviations in the value of the coupling parameter g_0. If we initialize the system in state $|e,0\rangle$, the transfer fidelity after time $T_\mathrm{t} = \pi/(2g_0)$ is

$$\mathcal{F}_\mathrm{t}(T_\mathrm{t}) = \frac{|g|^2}{\tilde{g}^2}\sin^2\left(\frac{\pi}{2}\frac{\tilde{g}}{g_0}\right), \tag{S2}$$

where $\tilde{g} = \sqrt{|g|^2 + \xi^2/4}$. Assuming that the deviations are small, $\xi \ll g_0$ and $|\epsilon| \ll 1$, we obtain

$$\tilde{g}/g_0 \approx 1 + \mathrm{Re}(\epsilon) + \frac{1}{2}\left[|\epsilon|^2 + \frac{\xi^2}{4g_0^2}\right], \tag{S3}$$

and the transfer fidelity can be approximated by

$$\mathcal{F}_\mathrm{t}(T_\mathrm{t}) \approx 1 - \frac{\xi^2}{4g_0^2} - \frac{\pi^2}{4}\left[\mathrm{Re}(\epsilon) + \frac{|\epsilon|^2}{2}\right]^2. \tag{S4}$$

When we restrict ourselves to frequency fluctuations with strength $\langle \xi^2 \rangle = \sigma^2$ only, Eq. (S4) reduces to Eq. (7) of the main text. The other contributions $\sim \epsilon$ are relevant to characterize other types of imperfections considered in Supplementary Note 3.

Supplementary Note 2: Error estimates

In its full form, the final correction Hamiltonian in Eq. (78) is cumbersome to treat. Supplementary Table I summarizes the numerical values for the most relevant coefficients for the sequences $\mathrm{XXYY}_{m=0}$, $\mathrm{XY8}_{m=2}$, and $X_{T/2}X_{3T/4}Y_{3T/4}Y_T$ [1], the latter denoted here as $\mathrm{XXYY}_\mathrm{GKLC}$. As we can see from the table, not all of the correction terms are significant and in certain limits, for example, when $\tau_\pi \to 0$, many of the corrections simplify further. Therefore, Eq. (78) is a convenient starting point to systematically evaluate the dominant effect of various different sources of imperfections for a given pulse sequence. In the following we perform such analysis in order to derive the scaling laws presented in the main text.

Resonant JC interactions

For the case of a single TLS, the last term in Eq. (78) does not appear. The interaction term of interest is determined by the coefficients $O^{(0)}_{x,y}$, while the parameters $O^{(0)}_z$ and $\vec{\Gamma}^{(0)}$ are zero for the sequences of interest. The remaining terms depend on either gT or ξT and can be suppressed by considering shorter interpulse spacings τ. In the following, we consider noise-induced and other errors separately.

Finite pulse spacing

The terms in Eq. (78) proportional to $\vec{\mathcal{G}}^{(0)}_2$ and $\vec{\mathcal{G}}^{(0)}_3$ are independent of ξ. They represent deviations from the ideal dynamics that arise from a finite spacing between the decoupling pulses. For all sequences of interest, $|\mathcal{G}^{(0)}_{2,z}| \gg |\mathcal{G}^{(0)}_{2,x,y}|, \mathrm{Re}(G^{(0)}_{3,u})$. Thus, in the absence of noise, the dominant source or errors arises from the correction given in Eq. (50). Using perturbation theory as outlined in Supplementary Note 4, we can evaluate its effect on a resonant state-transfer process and obtain a transfer error scaling as

$$\mathcal{E}_\mathrm{t} \approx 1.16 \times \frac{g^2 T^2 |\mathcal{G}^{(0)}_{2,z}|^2}{8|\eta|^2}. \tag{S5}$$

For the $\mathrm{XY8}_{m=2}$ sequence with $T = 8\tau$ this expression simplifies to $\mathcal{E}_\mathrm{t} \approx 1.88 g^2\tau^2$, while for the $\mathrm{XXYY}_{m=0}$ sequence with $T = 4\tau$ we obtain $\mathcal{E}_\mathrm{t} \approx 0.58 g^2\tau^2$. Compared to $\mathrm{XXYY}_{m=0}$ and $\mathrm{XY8}_{m=2}$, the $\mathrm{XXYY}_\mathrm{GKLC}$ sequence yields smaller values of $|\mathcal{G}^{(0)}_{2,z}|$, with $|\mathcal{G}^{(0)}_{2,z}| = 0$ in the limit of instantaneous pulses. In this case, the leading error is of higher order in $g\tau$ and is not captured by Eq. (78).

Noise cancellation

The condition $\vec{\Gamma}^{(0)} = 0$ ($\gamma_{x,y,z} = 0$ in the main text) ensures that the first term in Hamiltonian (68) vanishes up to lowest order in (ξT). However, at higher orders, terms in the Hamiltonian scaling as $\xi^2 T$ or $g\xi T$ still generate noise-induced deviations from the ideal dynamics. These corrections are determined by the coefficients $\vec{\Gamma}^{(1)}$ and $\vec{\mathcal{G}}^{(0)}_0$ and $\vec{O}^{(1)}$ and $\vec{\mathcal{G}}^{(0)}_1$, respectively. In the case of the $\mathrm{XXYY}_{m=0}$ sequence with finite pulse length $\tau_\pi = 0.1\tau$, we find that $\Gamma_\mathrm{pw} \equiv \Gamma^{(1)}_x = \Gamma^{(1)}_y \neq 0$, while the other coefficients are zero. This implies that the dominant effect of the noise stems from a contribution of the form

$$\tilde{H}_\mathrm{corr} \simeq \frac{\xi^2 T}{2}\Gamma_\mathrm{pw}(\sigma_x + \sigma_y). \tag{S6}$$

$\times 10^{-2}$	$\lvert O^{(0)}_{x,y}\rvert$	$\lvert \mathcal{G}^{(0)}_{2,z}\rvert$	$\lvert O^{(1)}_{x,y}\rvert$	$\Gamma^{(1)}_{x,y}$	$\lvert \mathcal{G}^{(1)}_{1,z}\rvert$	$\lvert \mathcal{G}^{(1)}_{2,z}\rvert$	$\mathrm{Re}(\mathcal{G}^{(1)}_{3,z})$	$\lvert O^{(2)}_{x,y}\rvert$	$\lvert \mathcal{G}^{(2)}_{2,z}\rvert$
$\mathrm{XXYY}_{m=0}$ ($\tau_\pi/\tau \to 0$)	50	25	0	0	0	0	-1.56	0.26	0.26
$\mathrm{XXYY}_{m=0}$ ($\tau_\pi/\tau = 0.1$)	50	25	0	0.38	0.19	0	-1.56	0.26	0.26
$\mathrm{XY8}_{m=2}$ ($\tau_\pi/\tau \to 0$)	45.02	20.26	0.77	0	0	0.69	-0.23	0.05	0.04
$\mathrm{XY8}_{m=2}$ ($\tau_\pi/\tau = 0.1$)	44.99	20.24	0.77	0	0	0.69	-0.23	0.05	0.04
$\mathrm{XXYY}_{\mathrm{GKLC}}$ ($\tau_\pi/\tau \to 0$)	50	0	18.75*	0	0	6.25	6.25	7.29*	0
$\mathrm{XXYY}_{\mathrm{GKLC}}$ ($\tau_\pi/\tau = 0.1$)	50	2.50	16.87*	0.38	0.07	5.06	4.77	5.95*	0.17

Supplementary Table I: Numerical values of the most relevant coefficients that determine the effective Hamiltonian given in Eq. (78) for the sequences $\mathrm{XXYY}_{m=0}$ and $\mathrm{XY8}_{m=2}$ introduced in Section "Dynamically protected vacuum Rabi oscillations", and the sequence $\mathrm{XXYY}_{\mathrm{GKLC}}$ introduced in Ref. [1]. For the first two sequences, $t_1 = \tau/2$. For all sequences, the parameters are evaluated in the limit of infinitely short pulses, $\tau_\pi/\tau \to 0$, and for a finite pulse length of $\tau_\pi/\tau = 0.1$. All other non-vanishing parameters are smaller than the ones listed in the table and do not contribute to the most relevant errors in leading order. We use "$*$" when $|O^{(1,2)}_x|$ and $|O^{(1,2)}_y|$ differ, in which case the larger value is given.

m	4	8	12	16	20	24	28	32	36	40
$\mathrm{Im}(\mathcal{G}^{(0)}_{uu}) \times (2\pi m)$	-1	-1	-1	-1	-1	-1	-1	-1	-1	-1
$\mathrm{Im}(\mathcal{G}^{(1)}_{uu}) \times (2\pi m) \times 10^2$	0	-3.98	0	-1.99	0	-1.33	0	-0.99	0	-0.80
$\mathrm{Im}(\mathcal{G}^{(2,1)}_{uu}) \times (2\pi m) \times 10^2$	-0.26	-0.10	-0.26	-0.22	-0.26	-0.24	-0.26	-0.25	-0.26	-0.25
$\mathrm{Im}(\mathcal{G}^{(2,2)}_{uu}) \times (2\pi m) \times 10^2$	-0.11	0.68	0.45	0.56	0.50	0.54	0.51	0.53	0.51	0.53

Supplementary Table II: Numerical values of the relevant coefficients that determine the noise-induced errors for a cavity mediated flip-flop process. The values are given for the $\mathrm{XY8}_m$ sequence discussed in Section "Imperfections", with $t_1 = \tau/2$ and pulse lengths $\tau_\pi/\tau \to 0$. Here, $u \in x, y$.

This correction induces the average transfer error given in Eq. (52), where we again followed the general approach in Supplementary Note 4 and used that $\langle \xi^4 \rangle = 3\sigma^4$.

In the case of the $\mathrm{XY8}_{m=2}$ sequence, we find that $\vec{O}^{(1)} \neq 0$, while all the other coefficients listed above are zero. In particular, the two contributions proportional to $\mathrm{Re}(O^{(1)}_x) \equiv O_1$ and $\mathrm{Im}(O^{(1)}_y) = -O_1$ in the second line of Eq. (68) will add up to a JC interaction with a modified coupling constant given in Eq. (51). By following the general derivation in Supplementary Note 1, we obtain

$$\mathcal{E}_{\mathrm{t}} \approx \left(\frac{\pi\sigma T}{2|\eta|} O_1\right)^2 = \left(\frac{2\sigma\tau}{3\pi}\right)^2, \tag{S7}$$

where for the second equality we have used that $O_1 = |\eta|/(6\pi^2)$. Interestingly, for the XY8 sequence, the residual error scales as σ^2 and it also doesn't vanish in the limit of infinitely fast π-rotations. This sequence is, however, more robust with respect to other types of pulse imperfections and may still be the preferred choice under most experimental conditions.

Finally, for the $\mathrm{XXYY}_{\mathrm{GKLC}}$ sequence we also find that $|O^{(1)}_{x,y}| \neq 0$, but these values are much larger than those found for the $\mathrm{XY8}_{m=2}$ sequence. This explains why the $\mathrm{XXYY}_{\mathrm{GKLC}}$ sequence performs worse than the other sequences in the presence of strong dephasing.

Protected spin-spin interactions

For the effective spin-spin interactions studied in the main text, the interaction terms of interest are determined by the coefficients $O^{(0)}_{x,y}$, the effective detuning Δ_{eff} and the coefficients $\mathcal{G}^{(0)}_{uv}$. For the following discussion we focus on the flip-flop interaction implemented by the same XY8 pulse sequence as described in Section "Imperfections". For values of the detuning that fulfill $\Delta = 2\pi m/T$ with $m = 4, 8, 12, ...$, we find that $O^{(0)}_{x,y} = 0$ [see Table in the main text] and $\mathrm{Im}(\mathcal{G}^{(0)}_{uu}) = -1/(2\pi m)$, and therefore, $J = g_1 g_2/\Delta$.

To investigate the effect of noise on the flip-flop interaction, we evaluate all parameters $\vec{\mathcal{W}}_j$, $\vec{\mathcal{O}}_j$, $\vec{\mathcal{G}}_{j,k}$ and $\mathcal{G}_{uv}$, and find that for the values of the detuning specified above the only non-zero parameters are $\mathcal{G}^{(1)}_{uu}, \mathcal{G}^{(2,1)}_{uu}$ and $\mathcal{G}^{(2,2)}_{uu}$ with $u \in x, y$. These corrections modify the effective coupling strength as $J \to J + \delta J$, where

$$\begin{aligned}\delta J \;=\;& \frac{J}{2}(\xi_1 + \xi_2) T\, \mathrm{Im}(\mathcal{G}^{(1)}_{uu})/\mathrm{Im}(\mathcal{G}^{(0)}_{uu}) \\ -\;& \frac{J}{4}(\xi_1^2 + \xi_2^2) T^2\, \mathrm{Im}(\mathcal{G}^{(2,1)}_{uu})/\mathrm{Im}(\mathcal{G}^{(0)}_{uu}) \\ -\;& \frac{J}{4}(\xi_1 \xi_2) T^2\, \mathrm{Im}(\mathcal{G}^{(2,2)}_{uu})/\mathrm{Im}(\mathcal{G}^{(0)}_{uu}).\end{aligned} \tag{S8}$$

For a single noise realization the corresponding error for the entanglement fidelity is then given by $\mathcal{E}_{\mathrm{e}} = \pi^2/4^2(\delta J/J)^2$. In Supplementary Table II we show the values of the relevant parameters for different m. Note that, while the value of $\mathrm{Im}(\mathcal{G}^{(0)}_{uu}) \times (2\pi m)$ is constant, the other parameters depend on m. For example, parameter $\mathrm{Im}(\mathcal{G}^{(1)}_{uu})$ is zero for $m = 4, 12, 20, \ldots$, but nonzero for $m = 8, 16, \ldots$. For $m \gg 1$, all these parameters converge to the values $\mathrm{Im}(\mathcal{G}^{(1)}_{uu})/\mathrm{Im}(\mathcal{G}^{(0)}_{uu}) \simeq 0$,

$\text{Im}(\mathcal{G}_{uu}^{(2,1)})/\text{Im}(\mathcal{G}_{uu}^{(0)}) \simeq 0.0025$ and $\text{Im}(\mathcal{G}_{uu}^{(2,2)})/\text{Im}(\mathcal{G}_{uu}^{(0)}) \simeq -0.005$. In this limit, the expression for δJ simplifies to

$$\delta J \approx -\frac{JT^2}{4^3 5^2}(\xi_1 - \xi_2)^2, \tag{S9}$$

and the average error $\mathcal{E}_{\rm e}$ for uncorrelated noise, where $\langle \xi_i^2 \xi_j^2 \rangle = (2\delta_{ij} + 1)\sigma^4$, is given by the result in Eq. (54).

Supplementary Note 3: Cavity decay

In this note we outline the derivation of an effective master equation for the reduced state of the TLSs, $\mu(t) = \text{Tr}_c\{\rho(t)\}$, where $\rho(t)$ is the full density operator obeying the master equation in Eq. (55). To do so we start with the JC Hamiltonian in the toggling frame, which we write as

$$\tilde{H}(t) = \Sigma(t)a^\dagger(t) + \Sigma^\dagger(t)a(t). \tag{S10}$$

Here, $a(t)$ is the cavity mode operator in the interaction picture and

$$\Sigma(t) = \sum_j \frac{g_j}{2}\left[f_x(t)\sigma_j^x - i f_y(t)\sigma_j^y\right], \tag{S11}$$

assuming ideal π-pulses for simplicity. Under the validity of the usual Born-Markov approximation, we can then follow the standard steps for the derivation of a master equation [2] and we obtain

$$\dot{\mu}(t) = -\int_0^t dt' \langle a(t)a^\dagger(t')\rangle_c [\Sigma^\dagger(t), \Sigma(t')\mu(t)] + \text{H.c.}, \tag{S12}$$

where $\langle a(t)a^\dagger(t')\rangle_c = e^{-(i\Delta+\kappa/2)(t-t')}$. In view of the periodicity of the DD sequence, it is more relevant to evaluate the change of the density operator over one period T. Therefore, we introduce the discrete time derivative

$$\left.\frac{\Delta\mu}{\Delta t}\right|_{t=nT} := \frac{\mu(nT+T) - \mu(nT)}{T} \tag{S13}$$

and take the formal limit $T \to 0$ and $\Delta\mu/\Delta t \to \dot{\mu}$ afterwards. This leaves us with the coarse-grained master equation

$$\begin{aligned}\dot{\mu} &= -\sum_{i,j}\sum_{\{u,v\}=\{x,y\}} \mathcal{K}_{uv}^{ij}[\sigma_i^u, \sigma_j^v \mu(t)] + \text{H.c.} \\ &\equiv -i\left(H_{\rm nh}\mu - \mu H_{\rm nh}^\dagger\right) + \mathcal{J}_{\rm rec}(\mu).\end{aligned} \tag{S14}$$

Here we have introduced the complex quantities

$$\begin{aligned}\mathcal{K}_{uv}^{ij} = \lim_{n\to\infty} \frac{g_i g_j}{4T} &\int_{nT}^{(n+1)T} dt \int_0^t dt' \\ &\times e^{-(i\Delta+\kappa/2)(t-t')} \tilde{f}_u^*(t)\tilde{f}_v(t'),\end{aligned} \tag{S15}$$

where $\tilde{f}_x(t) = f_x(t)$, $\tilde{f}_y(t) = -i f_y(t)$. By neglecting the recycling term $\mathcal{J}_{\rm rec}(\mu)$, the master equation in Eq. (S14) is equivalent to the evolution of the TLSs under the non-Hermitian Hamiltonian

$$H_{\rm nh} = -i\sum_{i,j}\sum_{\{u,v\}=\{x,y\}} \mathcal{K}_{uv}^{ij}\sigma_i^u\sigma_j^v. \tag{S16}$$

Thus, the real parts of the $\mathcal{K}_{uv}^{ij}$ correspond to effective loss rates, which reduce the norm of the wavefunction.

To evaluate the remaining integrals, we first calculate the integral over t'. To do so we write $\Delta = 2\pi m/T + \Delta_{\rm eff}$ and split the integral into a part up to time nT and the rest. For large enough n, such that $nT\kappa \gg 1$, we obtain

$$\begin{aligned}\int_0^t dt'\, e^{-(i\Delta+\kappa/2)(t-t')}\tilde{f}_v(t') &= \int_{nT}^t dt'\, \tilde{f}_v(t')e^{-(i\Delta+\kappa/2)(t-t')} \\ &+ \left(\frac{e^{-(i\Delta+\kappa/2)(t-nT)}}{1 - e^{-(i\Delta_{\rm eff}+\kappa/2)T}}\right)\int_0^T dt'\, \tilde{f}_v(t')e^{-(i\Delta+\kappa/2)(T-t')}.\end{aligned} \tag{S17}$$

After reinserting this result back into Eq. (S15) and combining all the terms, we end up with

$$\mathcal{K}_{uv}^{ij} = \frac{g_i g_j}{4}\left(\frac{T\tilde{\mathcal{J}}_{uv}(\kappa)}{1 - e^{-(i\Delta_{\rm eff}+\kappa/2)T}}\right), \tag{S18}$$

where

$$\tilde{\mathcal{J}}_{uv}(\kappa) = \int_0^T \frac{dt}{T}\int_{t-T}^t \frac{dt'}{T}\, e^{-(i\Delta+\kappa/2)(t-t')}\tilde{f}_u^*(t)\tilde{f}_v(t'). \tag{S19}$$

Discussion

For a single spin the decay part of the non-Hermitian Hamiltonian, $H_{\rm decay} = (H_{\rm nh} - H_{\rm nh}^\dagger)/2$, reduces to

$$H_{\rm decay} = -\frac{i}{2}\left(\gamma_{\rm eff}^e|e\rangle\langle e| + \gamma_{\rm eff}^g|g\rangle\langle g|\right), \tag{S20}$$

where we have introduced the effective decay rates

$$\gamma_{\rm eff}^{e/g} = 2\text{Re}\left\{\mathcal{K}_{xx} + \mathcal{K}_{yy} \pm i(\mathcal{K}_{xy} - \mathcal{K}_{yx})\right\}. \tag{S21}$$

To obtain a more meaningful expression, we consider the limit $|i\Delta_{\rm eff} + \kappa/2|T \ll 1$, in which case we can approximate $\tilde{\mathcal{J}}_{uv}(0) \simeq \tilde{\eta}_u^*\tilde{\eta}_\nu$, where $\tilde{\eta}_x = \eta_x$ and $\tilde{\eta}_y = -i\eta_y$. Therefore, for a pulse sequence with a non-vanishing first-order coupling, the main contribution to the decay rate is approximately given by

$$\gamma_{\rm eff}^{e/g} \approx \frac{g^2|\eta_x \pm \eta_y|^2\kappa}{4\Delta_{\rm eff}^2 + \kappa^2}. \tag{S22}$$

For example, for the case of an effective JC evolution with $\eta_x = \eta_y = \eta$, we recover the result $\gamma_{\rm eff}^g \approx 0$ and $\gamma_{\rm eff}^e \approx g_{\rm eff}^2\kappa/(\Delta_{\rm eff}^2 + \kappa^2/4)$, as expected for a weakly coupled JC model.

When the first-order coupling vanishes, i.e., $\eta_{x,y} = 0$, the corrections to $\tilde{\mathcal{J}}_{uv}(0)$ for finite κ must be taken into account. In this situation it is more convenient to express the $\mathcal{K}_{uv}^{ij}$ in terms of a Fourier series

$$\mathcal{K}_{uv}^{ij} = \frac{g_i g_j}{4} \sum_{n=-\infty}^{\infty} \frac{\left[\tilde{\eta}_n^{(u)}\right]^* \tilde{\eta}_n^{(v)}}{i(\Delta - 2\pi n/T) + \kappa/2}, \tag{S23}$$

where

$$\tilde{\eta}_n^{(u)} = \frac{1}{T}\int_0^T dt\, \tilde{f}_u(t) e^{i2\pi n t/T}. \tag{S24}$$

For the case of a single TLS we obtain

$$\gamma_{\rm eff}^{e/g} = \frac{g^2\kappa}{4} \sum_{n=-\infty}^{\infty} \frac{|\eta_n^\pm|^2}{(\Delta - 2\pi n/T)^2 + \kappa^2/4}, \tag{S25}$$

where

$$\eta_n^\pm = \frac{1}{T}\int_0^T dt\, \left[f_x(t) \pm f_y(t)\right] e^{i2\pi n t/T}. \tag{S26}$$

From this expression we immediately see that in the limit of very large detuning, $\Delta T \to \infty$, the two rates for the ground and the excited state are approximately the same and given by

$$\begin{aligned}\gamma_{\rm eff}^{e/g} &\approx \frac{g^2}{4\Delta^2}\kappa \sum_n |\eta_n^\pm|^2 \\ &= \frac{\gamma_0}{4}\frac{1}{T}\int_0^T dt\, \left[f_x(t) \pm f_y(t)\right]^2 = \frac{\gamma_0}{2},\end{aligned} \tag{S27}$$

with $\gamma_0 = (g/\Delta)^2\kappa$. The last equality in Eq. (S27) holds for sequences with $\int_0^T dt\, f_x(t) f_y(t) = 0$, which is fulfilled for all noise-canceling sequences. Note that for the undriven case $f_x(t) = f_y(t) = 1$ and we recover the usual result $\gamma_{\rm eff}^g = 0$ and $\gamma_{\rm eff}^e \approx \gamma_0 = g^2\kappa/\Delta^2$.

As illustrated in Supplementary Figure 1 for the XY8 sequence, the dependence of the effective decay rates for intermediate values of Δ is more involved and exhibits multiple resonances. These resonances occur whenever $\Delta \approx 2\pi m/T$ and $\eta_m^\pm \neq 0$, in which case $\gamma_{\rm eff}^{e/g}$ will again be approximately given by Eq. (S22) (note that $\eta_x \pm \eta_y = \eta_m^\pm$). On the contrary, if around the m-th harmonic, $\eta_m^\pm = 0$, then $\gamma_{\rm eff}^{e/g}$ will be determined by the residual sum

$$\gamma_{\rm eff}^{e/g} \approx \frac{g^2\kappa}{4\Delta^2} \sum_{n\neq m} \frac{|\eta_n^\pm|^2}{(1 - n/m)^2}, \tag{S28}$$

which, up to numerical prefactors, is comparable to γ_0.

Note that in the case of $N = 2$ and $g_1 \simeq g_2$ also the fact that both TLSs couple to the cavity mode symmetrically must be taken into account. In the far-detuned regime, the effective decay is then determined by the non-Hermitian Hamiltonian

$$H_{\rm decay} = -i\frac{g^2\kappa}{2\Delta^2}\left(\mathbb{1} + \sigma_1^+\sigma_2^- + \sigma_1^-\sigma_2^+\right). \tag{S29}$$

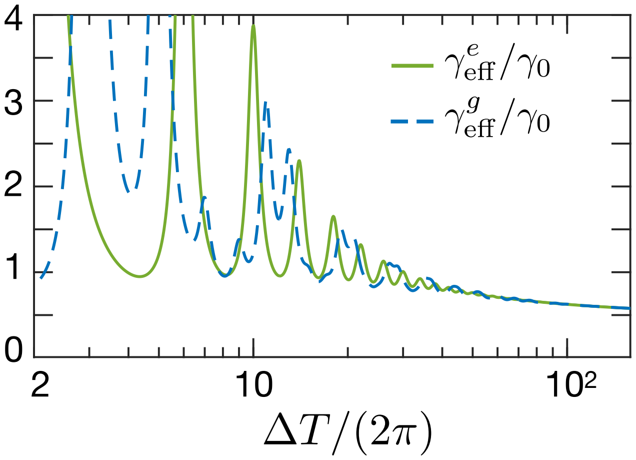

Supplementary Figure 1: Plot of the effective decay rates $\gamma_{\rm eff}^{e/g}$ as a function of the cavity detuning Δ for a single TLS driven by a XY8 sequence with $t_1 = \tau/2$ and $\tau_\pi = 10^{-2}\tau$.

Within the subspace $\{|eg\rangle, |ge\rangle\}$, this leads to the same decay dynamic as for the case of two undriven TLSs. Therefore, also the same effective decay rates appear in the expressions for the entanglement fidelities in Eq. (59) and Eq. (62).

Supplementary Note 4: Perturbation theory for the JC state transfer

To calculate the transfer error for the JC Hamiltonian, we first identify the effective Hamiltonian term responsible for the error $\tilde{H}_{\rm err}$, with coupling ϵ. The total effective Hamiltonian is then $\tilde{H} = \tilde{H}_{\rm int}^{\rm eff} + \tilde{H}_{\rm err}$, where $\tilde{H}_{\rm int}^{\rm eff} = \eta g\left(\sigma_- a^\dagger e^{i\phi} + {\rm H.c.}\right)$ is the ideal effective Hamiltonian. For small errors $\epsilon T_{\rm t} \ll 1$, the effective evolution operator $e^{-i\tilde{H}T_{\rm f}}$ can be approximated using the Dyson series as

$$U \approx U_{\rm t}\Big\{1 - i\int \tilde{V}(t) - \iint \tilde{V}(t)\tilde{V}(s)\Big\}, \tag{S30}$$

where $U_{\rm t} = e^{-i\tilde{H}_{\rm int}^{\rm eff}T_{\rm t}}$, $\tilde{V}(t) = e^{i\tilde{H}_{\rm int}^{\rm eff}t}\tilde{H}_{\rm err}e^{-i\tilde{H}_{\rm int}^{\rm eff}t}$ and $\int \equiv \int_0^{T_{\rm f}} dt$ and $\iint \equiv \int_0^{T_{\rm f}} dt \int_0^t ds$ for clarity. The state fidelity between the ideal final state $U_{\rm t}|\psi_0\rangle$ and the perturbed final state $U|\psi_0\rangle$ is then $\mathcal{F} = 1 - \mathcal{E}$, where

$$\mathcal{E} \approx 2\iint \langle\psi_0|\tilde{V}(t)\tilde{V}(s)|\psi_0\rangle - \left[\int \langle\psi_0|\tilde{V}(t)|\psi_0\rangle\right]^2. \tag{S31}$$

[1] P. Groszkowski, M. Koppenhöfer, H.-K. Lau, and A. A. Clerk, Reservoir-engineered spin squeezing: Macroscopic even-odd effects and hybrid-systems implementations, Phys. Rev. X **12**, 011015 (2022).

[2] C. W. Gardiner and P. Zoller, *Quantum Noise* (Springer, Berlin, 2004).
